# Synergy-Guided Molecular Redesign of Predicted P‑Glycoprotein Substrates toward Synthetically Feasible Non-Substrates

**DOI:** 10.1021/acs.jcim.6c01938

**Published:** 2026-10-19

**Authors:** Soyeon Jeon, Sung Wook Moon, Hyun Woo Kim

**Affiliations:** † Department of Chemistry, 65419 Gwangju Institute of Science and Technology, Gwangju 61005, Republic of Korea; ‡ Center for Quantum Technology, Korea Institute of Science and Technology, Seoul 02792, Republic of Korea

## Abstract

Drug efflux mediated by P-glycoprotein (P-gp) remains a major barrier to effective small-molecule therapeutics, limiting both bioavailability and central nervous system exposure. While machine learning models can predict P-gp substrates, translating these predictions into actionable molecular design remains largely unexplored, particularly in enabling the rational conversion of substrates into nonsubstrates to mitigate efflux liability. Here, we introduce the synergy-guided fragment genetic algorithm (SF-GA), a computational framework that transforms predictive insight into an interpretable molecular design strategy. By integrating fragment synergy analysis into the mutation stage of a genetic algorithm, SF-GA biases fragment replacement toward co-occurring substructures associated with nonsubstrate classification, enabling interpretability at the chemical structure level and property-directed molecular optimization. Compared with genetic-algorithm and reinforcement-learning baselines under a fixed function-evaluation budget, SF-GA achieves competitive optimization performance while producing molecules with the highest retrosynthetic accessibility among all methods tested. Unlike the baselines, every accepted mutation is reported together with the discarded and inserted fragments and their synergy scores, providing a fragment-level rationale for each structural change; ablation against an unguided and an importance-only control isolates the contribution of bit co-occurrence. The method enables scaffold-preserving and similarity-constrained editing that converts P-gp substrates into predicted nonsubstrates. Docking against two human P-gp structures reveals weaker predicted binding for molecules with higher predicted nonsubstrate probability, motivating future experimental evaluation of P-gp transport.

## Graphical abstract

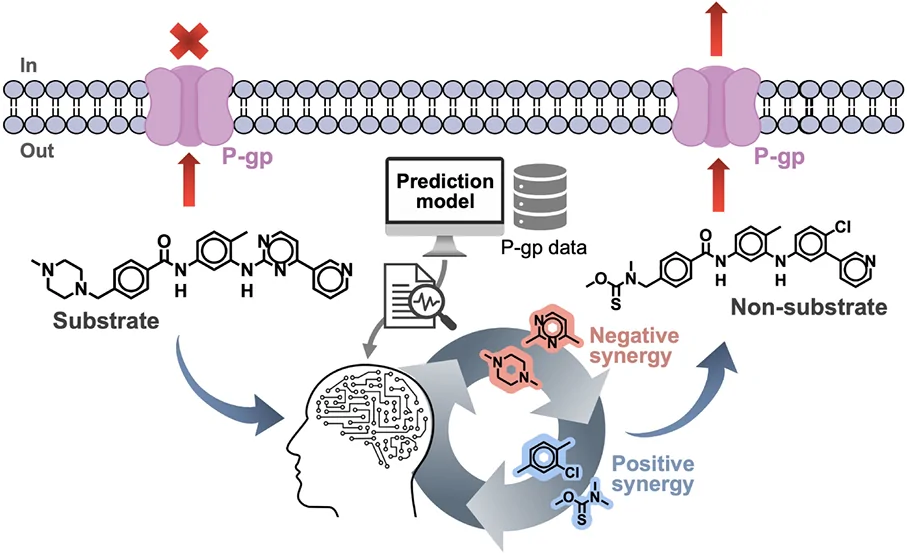

## 1 Introduction

Multidrug resistance^1,2^ remains a major challenge in drug discovery and development and is largely driven by P-glycoprotein (P-gp)^3^ that actively effluxes structurally diverse small molecules. P-gp is widely expressed in tissues and physiological barriers, most notably the blood–brain barrier (BBB),^3^ where it limits drug entry into the brain by exporting a broad range of substrates.^4,5^ Consequently, when brain-targeting drugs act as P-gp substrates, their therapeutic efficacy is reduced due to this active efflux from the central nervous system (CNS).^6–8^ Although inhibition of P-gp-mediated efflux can enhance substrate bioavailability and BBB penetration,^9^ indiscriminate inhibition or modulation of P-gp at the BBB often leads to toxicity,^10^ as P-gp plays a critical role in cellular protection by removing toxins.^11^ While P-gp-mediated efflux is advantageous for restricting the CNS entry of peripheral drugs, minimizing substrate liability remains a prerequisite for successful CNS drug discovery. Given the clinical challenges of direct transporter modulation, strategic molecular design aimed at circumventing P-gp recognition offers a more robust path toward improving brain penetration.

Recent advances in machine learning have significantly improved the predictive performance of models for classifying P-gp substrates.^12,13^ Several models have been employed, including support vector machines (SVM), naive Bayesian classifiers,^14^ decision trees,^15^ and graph neural networks based on AttentiveFP.^16^ In addition, the molecular dynamics fingerprint (MDFP) approach, which leverages molecular dynamics simulations, has been investigated.^17^ A multimodal contrastive learning framework was developed for P-gp substrate prediction, reporting superior classification performance.^18^ Both classical and deep learning models have demonstrated strong predictive capabilities in distinguishing P-gp substrates from nonsubstrates.^12,16^ In parallel, some studies have focused on predicting the transport potential of P-gp substrates. For example, a gradient boosting–based model was developed for predicting P-gp efflux potential.^19^ Moreover, P-gp substrate prediction has been incorporated into models that consider BBB permeability and other absorption, distribution, metabolism, excretion, and toxicity (ADMET)-related properties.^20^ Automated machine learning approaches have also been employed to construct multi-end point ADMET predictors, with P-gp substrate classification included as a specific evaluation task.^21^

With advances in predictive performance, recent studies have explored integrating P-gp substrate classification within generative models for drug design.^22,23^ Deep molecular generative models include generative adversarial networks (GANs), variational autoencoders (VAEs), and diffusion models.^24–26^ These approaches can be considered either as distribution learning methods, which model the training data distribution, or as goal-directed optimization methods that explicitly optimize user-defined objectives.^22,27^ In goal-directed generation, property predictors are coupled to generators as scoring functions or constraints to bias sampling toward desired profiles, enabling the direct optimization of ADMET end points, including transporter-associated tasks such as P-gp substrate classification.^27–29^ Predictor-guided objectives in reinforcement learning frameworks expanded the exploration beyond local analogs.^30^ Fragment-based hierarchical action spaces combined with synthesizability-guided action masks can perform Pareto optimization over drug-like objectives, integrating feasibility considerations into goal-directed generation.^31^ In addition, postgeneration ADMET profiling workflows were developed to include assessment of P-gp substrate classification.^32^

In addition to deep generative models, evolutionary optimization methods such as genetic algorithms (GAs) have been studied for exploring large chemical spaces and optimizing composite scoring functions.^33–35^ GAs operate through iterative cycles of mutation and selection and can directly incorporate predicted scores from property models, including P-gp substrate classifiers, into multiobjective fitness functions.^36^ However, efficient exploration of the chemical search space remains a major challenge when applying GAs to molecular design.^37^ Consequently, recent studies have focused on improving exploration strategies within evolutionary frameworks. The graph-based genetic algorithm (GB-GA) mutates molecular graphs and selects candidate molecules according to predicted property scores.^38^ This strategy has been extended by recording mutation histories and structural transitions during evolutionary exploration, improving the interpretability of optimization trajectories.^39^ Uncertainty-controlled GAs integrate ensemble predictors with evolutionary optimization under multiple objectives to mitigate sensitivity to model uncertainty.^40^ To improve the synthesizability of generated molecules, molecular editing can be constrained using predefined reaction rules to explore reaction-compatible chemical space.^41^ A fragment-based evolutionary algorithm recombines fragments partitioned by gene types, guiding the search toward building blocks compatible with practical synthesis.^42^

Despite advances in predictive and generative models, their black-box nature remains a major concern in molecular design.^43,44^ To address this issue, explainable artificial intelligence (XAI) methods such as SHapley Additive exPlanations (SHAP)^45^ and Local Interpretable Model-agnostic Explanations (LIME)^46^ have been applied to molecular property prediction to identify influential atoms, fragments, or substructures and reveal model dependence on specific chemical patterns.^47–50^ For example, a SHAP-based synergistic group analysis was proposed to characterize cooperative effects among molecular substructures in BBB permeability prediction, demonstrating how interaction-level explanations complement single-feature attributions.^51^ In P-gp substrate classification, integrated gradients and attention mechanisms have also been used to highlight structural determinants driving model outputs.^16^ More broadly, multilevel interpretability has been explored to explain model behavior across molecular, fragment, and atom-level representations. However, these predictor-side interpretability approaches remain limited for molecular design, where explanations should connect model outputs to chemically meaningful transformation choices.

Even when such predictor-level explanations are available, a gap often remains between model outputs and practical decision-making in lead optimization, where medicinal chemistry relies on iterative structure–activity relationship (SAR) learning and explicit structural modifications.^52,53^ In this context, the actionable unit is typically a sequence of chemically interpretable transformations that can be tested and prioritized for synthesis and assay. Accordingly, attribution maps or feature-importance scores may be insufficient when they fail to translate into concrete transformation strategies for improving multiobjective profiles such as potency and ADMET properties.^54^ This limitation is more evident in foundation model–based generation, where latent representations obscure the connection from design goals through intermediate modifications to final molecules, making it difficult to derive interpretable modification sequences for P-gp substrate classification.^55^ These challenges motivate approaches that integrate accurate predictors with optimization procedures to generate interpretable paths controlling P-gp substrate probability.

To this end, we propose the synergy-guided fragment genetic algorithm (SF-GA), a new molecular editing framework for transforming P-gp substrates into nonsubstrates at the fragment level, and vice versa. SF-GA explores the vast chemical space efficiently by inducing mutations according to synergistic group analysis. Specifically, by quantifying synergistic scores for fragment replacements, SF-GA enables goal-oriented property transformations and provides explanations throughout the generation process. Thus, SF-GA reduces the black-box nature of generative models by providing a transparent optimization pathway during molecular editing. By utilizing synthetically accessible fragments, SF-GA can offer the synthetic feasibility of P-gp substrates or nonsubstrates. This approach is relevant to late-stage drug development, where synthetic feasibility and target properties should be considered together. Although this work focuses on P-gp substrates, the method can be applicable to other molecular optimization tasks involving structure-dependent properties.

## 2 Computational Details

### 2.1 Data Preparation

The data set containing P-gp substrates and nonsubstrates was obtained from a previous study.^21^ This data set was compiled from several sources, including ChEMBL^56,57^ and Metrabase,^58^ and consists of 1,598 P-gp substrates and 1,405 P-gp nonsubstrates. All molecules were provided in simplified molecular input line entry system (SMILES) format, then standardized and processed using RDKit.^59^ Compounds that failed sanity checks or had duplicate canonical SMILES were excluded from further analysis. During data preparation, we also analyzed the chemical space and Lipinski properties^60^ of the data set to consider potential bias toward specific chemical profiles in Figure S1.

### 2.2 Predictive Model Construction and Interpretation

A P-gp nonsubstrate prediction model, hereafter referred to as MACCS-XGBoost, was trained using eXtreme Gradient Boosting (XGBoost)^61^ with molecular access system (MACCS) fingerprints^62^ as input features. The data set was partitioned into training and test sets using a 9:1 ratio, and all reported metrics are averaged over five independent runs on the test set. In this work, the MACCS-XGBoost model achieved the highest ROC-AUC of 0.883, outperforming support vector machine,^63^ random forest,^64^ deep neural network, graph convolutional network,^65^ and AttentiveFP,^66^ as listed in Table S1 (see the Supporting Information). Thus, we adopted the XGBoost model for further analysis. The molecules were encoded using MACCS keys, a 167-bit binary fingerprint vector representing predefined chemical substructures.^62^ In MACCS keys, each bit corresponds to an interpretable chemical pattern, enabling direct mapping between model features and molecular substructures. We note that MACCS is a low-dimensional fingerprint representation. Nevertheless, among the structural fingerprints evaluated using XGBoost, MACCS keys achieved the highest ROC-AUC (0.883), outperforming ECFP4,^67^ PubChem,^68^ and RDKit fingerprints^69^ (Table S2). In addition, MACCS was selected because each bit corresponds to a predefined, human-readable chemical pattern, which enables the substructure-level interpretation described in Section 2.3.

To obtain quantitative feature importance values from the predictive model, SHAP^45^ was applied to the trained XGBoost model. SHAP analysis provides feature importance as

1 $$\phi_{i}=\sum_{S\subseteq N\backslash \{i\}}\frac{\left|S\right|!\left(|N|-|S|-1\right)!}{\left|N\right|!}\left[f\left(S\cup \left\{i\right\}\right)-f\left(S\right)\right]$$

Here, ϕ_*i*_ denotes the SHAP value, representing the contribution of feature *i* to the model output. *f*(*S*) denotes the output of the trained machine learning model conditioned on the feature subset *S*, where missing features are marginalized over the background distribution. *N* represents the full set of input features, and *S*⊆*N*\{*i*} denotes a subset excluding feature *i*. The term *f*(*S*∪{*i*}) corresponds to the model output when feature *i* is included in subset *S*. The weighting factor accounts for all possible feature coalitions, ensuring that the marginal contribution of feature *i* is averaged across all subsets. Each feature corresponds to a MACCS fingerprint bit representing a predefined chemical substructure. Accordingly, SHAP values quantify the contribution of individual structural motifs to the predicted probability of being a P-gp nonsubstrate. In this work, TreeExplainer from the SHAP framework^70^ was used to perform SHAP analysis on the XGBoost model.

### 2.3 Synergy Group Analysis

We adopted a substructure-based framework to capture synergistic effects between molecular fragments following Lee et al.^51^ In this approach, molecules were first grouped according to the SHAP contribution of each fingerprint bit, distinguishing those with strong positive contributions from those with weak contributions. For each bit, aggregated fingerprint profiles of the two groups were compared, and their difference was computed to obtain a vector representing co-occurring substructures associated with that bit. Substructure combinations supporting the prediction of the P-gp nonsubstrate class were defined as the supporting synergistic group (SG), whereas those associated with the substrate class were defined as the opposing synergistic group (OG). Substructure combinations with difference-vector values exceeding one standard deviation from the distribution mean were classified as SG for positive deviations and OG for negative deviations. The term “synergy” in this work refers to a heuristic for identifying feature associations^51^ rather than a formal interaction term or a description of P-gp transport.

### 2.4 BRICS-Based Fragment Library

To improve synthesizability, the mutation step of SF-GA employs fragments generated using the building blocks for rapid interactive construction of molecular structures (BRICS) method,^71^ as BRICS is a systematic fragmentation approach that cleaves bonds and assigns connection-point labels according to retrosynthetic rules.

For BRICS-based fragment mutation, a fragment library was constructed by categorizing fragments according to their connectivity using the ZINC 250K data set.^72^ Fragments with a single attachment point were initially classified as arms, representing terminal groups, whereas fragments with two or more attachment points were designated as linkers, serving as scaffolds connecting molecular substructures. In the subsequent step, fragments were organized by connection-point label, referred to as fragment type. Finally, the fragment library was structured according to attachment point count and fragment type to facilitate efficient retrieval. The library was then filtered by excluding fragments matching PAINS,^73^ Brenk,^74^ or NIH^75^ filters. This filtering reduces the likelihood of generating undesirable molecules.

### 2.5 Synergy-Guided Fragment Genetic Algorithm (SF-GA)

SF-GA generates candidate molecules by optimizing a desired property, such as the P-glycoprotein (P-gp) nonsubstrate probability, through iterative, fragment-level mutations. Each generation consists of four sequential steps: (1) fragment targeting, (2) fragment replacement, (3) candidate evaluation, and (4) molecule selection (Figure 1b). The initial two steps constitute the mutation step, and during this stage, each parent molecule is decomposed into BRICS fragments that contain connection-point labels indicating specific attachment points. Fragments for replacement are stochastically selected from a BRICS-based fragment library. The overall workflow is described in Figure 1.

**Figure 1:**
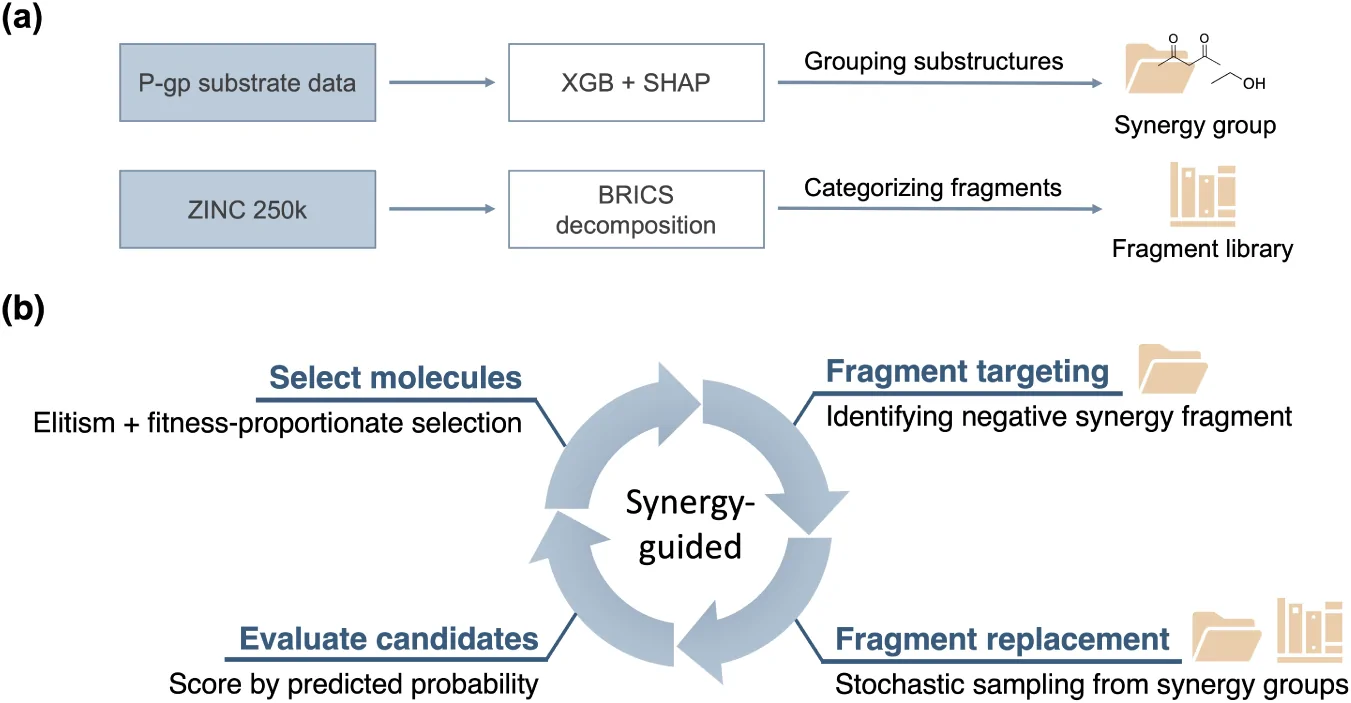
SF-GA workflow. (a) Preparation steps for the SF-GA. (b) Genetic algorithm workflow, where each step is represented by icons.

#### 2.5.1 Synergy-Guided Fragment Scoring

To enable more interpretable mutation, a fragment-level synergy score is defined based on synergy groups. During the mutation step, each parent molecule is evaluated using this scoring scheme.

Consider binary fingerprint vectors β_*p*_ ∈ {0, 1}^*d*^ for the parent molecule and β_*f*_ ∈ {0, 1}^*d*^ for the fragment, where *d* = 167 is the dimension of the MACCS keys. Let **G** ^(SG)^, **G** ^(OG)^ ∈ {0, 1}^*d* × *d*^ denote the synergy-group assignment matrices for supporting synergy groups (SGs) and opposing synergy groups (OGs), respectively, where **G**_*ij*_ = 1 indicates that fragment bit *j* belongs to the synergy group associated with parent-fingerprint bit *i*, and 0 otherwise. The numbers of supporting and opposing interactions are defined as

2 $$N_{\mathrm{SG}}=\beta_{p}^{T}G^{\left(\mathrm{SG}\right)}\beta_{f},,N_{\mathrm{OG}}=\beta_{p}^{T}G^{\left(\mathrm{OG}\right)}\beta_{f}$$

These quantities correspond to the numbers of supporting and opposing pairwise co-occurrences between the active bits in the parent fingerprint and fragment bits. The synergy score is then defined as

3 $$Z\left(\beta_{p},\beta_{f}\right)=\{\begin{array}{ll} \frac{N_{\mathrm{SG}}-N_{\mathrm{OG}}}{N_{\mathrm{SG}}+N_{\mathrm{OG}}}, & \mathrm{if}N_{\mathrm{SG}}+N_{\mathrm{OG}}>0,\\ 0, & \mathrm{otherwise} \end{array}$$

The resulting metric is bounded within the interval [−1, 1] and changes sign when supporting and opposing interactions are exchanged. Based on the scoring procedure described above, the SF-GA is performed as follows.

- **Step 1: Fragment Target Identification** BRICS fragments of the parent molecule are evaluated using the synergy score *Z*(β_*p*_, β_*f*_), where β_*p*_ and β_*f*_ denote the binary fingerprint vectors of the parent molecule and fragment, respectively. A threshold (*T* = μ_–_ – σ_–_) is used to prioritize fragments with lower synergy scores during sampling in this work. Here, μ_–_ and σ_–_ denote the mean and standard deviation of the negative synergy scores, respectively. If no fragment satisfies the threshold criterion, fragments with negative synergy scores are sampled. In the absence of such fragments, all fragments are considered for sampling.
- **Step 2: Fragment Replacement** Targeted fragments are replaced by sampling from a candidate pool retrieved from the fragment library. A stringent threshold (*T* = μ_+_ + σ_+_) is initially applied to prioritize high-synergy fragments, and is then relaxed to *Z* > 0 if the pool becomes empty. Here, μ_+_ and σ_+_ denote the mean and standard deviation of the positive synergy scores, respectively. When no positive synergy scores are observed, all fragments are considered for sampling. Offspring are subsequently assembled via BRICS recombination. The coefficients applied to the standard deviations in Steps 1 and 2 are treated as user-defined hyperparameters. Increasing these coefficients increases the predicted nonsubstrate probability per generation while reducing the candidate pool (Table S3 and Figure S2). Therefore, *T* = μ_–_ – σ_–_ and *T* = μ_+_ + σ_+_ were used throughout this work as a compromise between selectivity and pool size.
- **Step 3: Candidate Evaluation** Generated molecules were standardized and filtered for chemical validity and uniqueness using RDKit.^59^ To improve the robustness of molecular evaluation and reduce dependence on a single molecular representation, candidate molecules were assessed using an ensemble prediction model. The ensemble comprised three XGBoost^61^ predictors trained on MACCS keys,^62^ ECFP4 fingerprints,^67^ and RDKit descriptors.^59^ It also included an AttentiveFP^66^ predictor, which learns graph-based molecular representations through graph attention. Candidates were classified using a decision threshold of 0.540, determined by the Youden J statistic^76^ from the ensemble ROC curve, rather than the conventional threshold of 0.5 (Table S4). The fitness of each candidate was defined as the predicted probability of being a P-gp nonsubstrate, *p̂*_nonsubs_, obtained from the ensemble model.
- **Step 4: Molecule Selection** The next generation is formed using a dual strategy: an elitist approach preserves the *N* highest ranking scaffolds to ensure exploitation, while fitness proportionate selection draws an additional *N* molecules to promote exploration of the chemical space. In this work, *N* was set to 100.

## 3 Results and Discussion

### 3.1 Goal-Directed Optimization of P-gp Non-Substrates

The goal of this work is to optimize molecules toward the P-gp nonsubstrate property using SF-GA, a synergy-guided structural editing approach. To evaluate goal-directed optimization, SF-GA was compared with representative molecular optimization methods. The baselines were REINVENT^77^ and three GAs implemented in the GuacaMol benchmark framework,^78^ namely GB-GA,^38^ FragGT,^42^ and SMILES-GA.^79^ Molecular optimization is often performed from individual compounds rather than populations. Accordingly, we additionally evaluated SF-GA without the selection step. Each of the 100 starting molecules was optimized independently, with its offspring propagated only from the corresponding starting molecule and without competition or replacement among molecules. The evaluation budget and all other components were unchanged. All methods were assessed using the practical molecular optimization (PMO) benchmark,^80^ which measures the area under the curve (AUC) of the top-*K* average property value as a function of the number of function evaluations, capturing both optimization performance and sample efficiency. For a fair comparison, all GA-based methods used the same initial pool of 100 randomly selected P-gp substrates in each independent run. Each function evaluation corresponded to an evaluation by the ensemble predictor described in Section 2.5, which was used consistently across all methods. All methods generated 200 molecules per optimization step, corresponding to the offspring size used in SF-GA.

Model performance was evaluated in terms of optimization efficiency and the quality of the generated molecular set. Optimization efficiency was quantified as the AUC of the top-50 average *p̂*_nonsubs_ as a function of the number of function evaluations, with the budget limited to 5,000 evaluations. The quality of the generated molecules was evaluated using quantitative estimate of drug-likeness (QED),^81^ synthetic accessibility score (SAscore),^82^ synthetic Bayesian accessibility (SYBA),^83^ retrosynthetic accessibility score (RAscore),^84^ molecular weight (MW), and internal diversity.^85^ Higher QED values indicate greater drug-likeness and lower SAscore values indicate greater synthetic accessibility. SYBA and RAscore assess synthesizability from complementary perspectives, using a Bayesian classifier and the likelihood of successful retrosynthetic planning, respectively. Internal diversity measures the structural variety within the generated molecules, reflecting the ability of each method to explore the chemical space. Novelty was defined as 1 – *S*_NN_, where *S*_NN_ is the maximum ECFP4 Tanimoto similarity between a generated molecule and molecules in the training set.

As shown in Figure 2 and Table 1, GB-GA showed the highest mean AUC top-50 (0.965), and REINVENT showed a similar value (0.964). REINVENT showed the best mean performance in QED, SAscore, SYBA, and internal diversity, whereas SF-GA achieved the highest mean RAscore (0.932) with competitive SAscore and SYBA values. REINVENT showed strong performance on these metrics, consistent with its reinforcement learning-based optimization of a model pretrained on the ChEMBL database. Since the GA-based methods started from an initial pool of 100 experimentally known P-gp substrates, evaluations of these initial molecules were counted toward the function evaluation budget, leaving fewer evaluations for subsequent optimization. Nevertheless, SF-GA achieved comparable optimization performance while exhibiting higher novelty than REINVENT. As shown in Figures S4 and S5, most molecules generated by SF-GA remained within the chemical distribution of the P-gp data set while extending toward neighboring chemical regions. These results suggest that SF-GA can increase molecular novelty while maintaining proximity to the chemical space represented by the training data.

**Table 1:** Performance of SF-GA and Baseline Models on the PMO Benchmark^a^

Metric	SF-GA	SF-GA (selection off)	REINVENT	GB-GA	FragGT	SMILES-GA
AUC top-50	0.948 ± 0.004	0.945 ± 0.004	0.964 ± 0.001	0.965 ± 0.002	0.955 ± 0.004	0.872 ± 0.024
*p̂*_nonsubs_	0.992 ± 0.001	0.988 ± 0.001	0.982 ± 0.001	0.996 ± 0.001	0.994 ± 0.000	0.971 ± 0.011
QED	0.431 ± 0.044	0.318 ± 0.055	0.603 ± 0.026	0.289 ± 0.085	0.345 ± 0.071	0.197 ± 0.090
SAscore	2.962 ± 0.159	3.168 ± 0.146	2.538 ± 0.084	4.523 ± 0.297	3.257 ± 0.334	6.444 ± 0.549
SYBA	40.9 ± 10.4	40.8 ± 9.1	48.6 ± 5.4	–17.0 ± 15.2	–1.6 ± 14.7	–53.5 ± 31.4
RAscore	0.932 ± 0.037	0.857 ± 0.041	0.900 ± 0.029	0.754 ± 0.146	0.888 ± 0.107	0.479 ± 0.309
MW	359.3 ± 38.9	476.4 ± 45.6	266.7 ± 11.4	220.8 ± 37.9	257.6 ± 66.2	574.5 ± 175.0
Diversity	0.744 ± 0.041	0.776 ± 0.016	0.877 ± 0.007	0.697 ± 0.064	0.682 ± 0.094	0.756 ± 0.094
Novelty	0.721 ± 0.016	0.740 ± 0.010	0.665 ± 0.016	0.806 ± 0.029	0.758 ± 0.027	0.820 ± 0.044
CPU time (min/core)	4.16	5.34	8.15	1.98	2.65	7.88

a AUC top-50 denotes the area under the curve of the top-50 average p̂ non-subs as a function of the number of function evaluations, normalized to a budget of 5,000 evaluations. All remaining metrics were computed from the top-50 molecules at the end of the budget. Values are reported as mean ± standard deviation over 10 independent runs.

**Figure 2:**
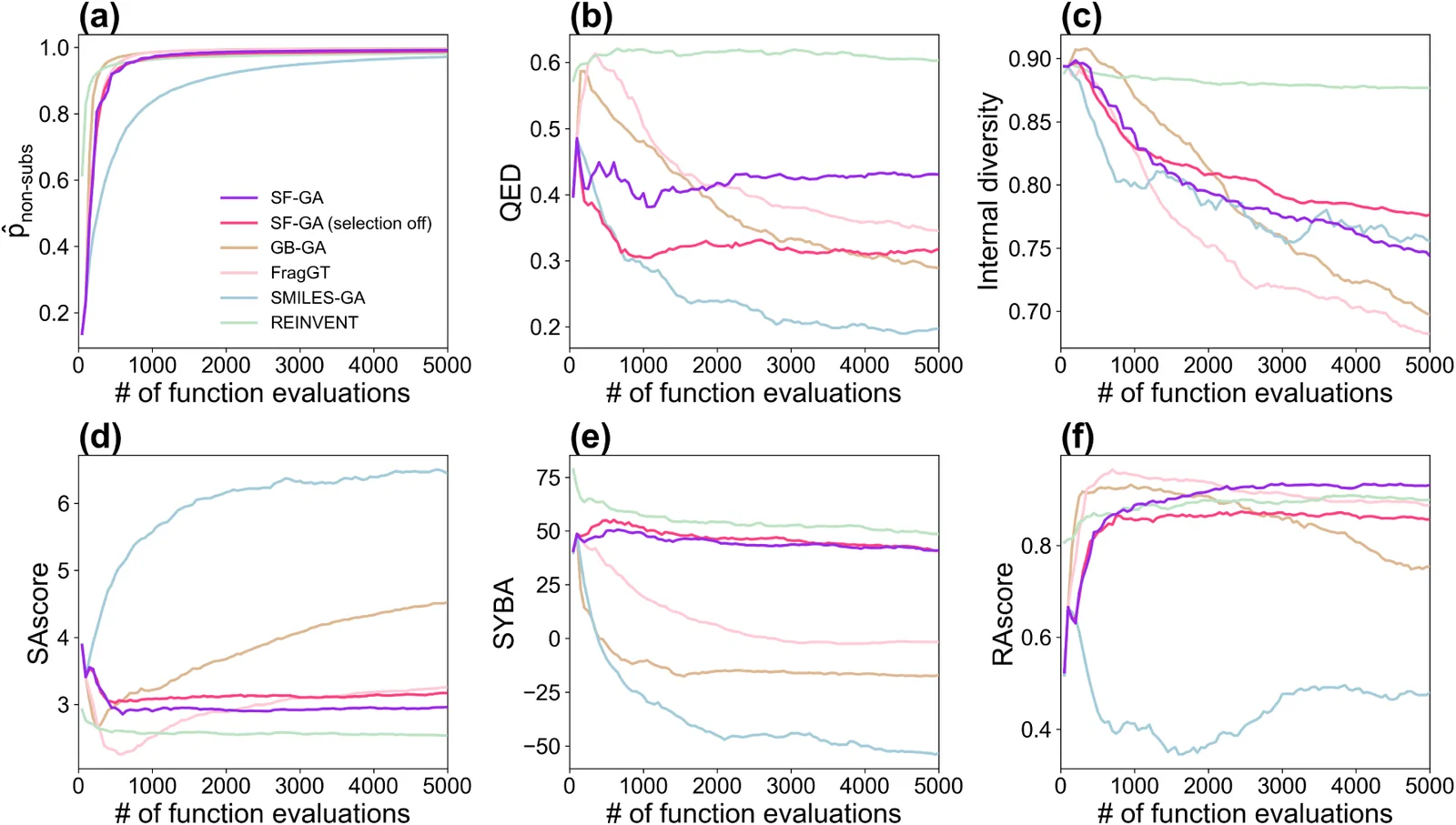
Performance comparison of molecular optimization methods. Optimization trajectories with respect to the number of function evaluations for (a) *p̂*_nonsubs_, (b) QED, (c) internal diversity, (d) SA score, (e) SYBA, and (f) RA score. Curves represent the mean values over 10 independent runs.

A potential advantage of SF-GA lies in its BRICS-constrained replacement, which enables structural expansion through chemically constrained fragment recombination. This approach may facilitate the exploration of larger molecular structures while maintaining relatively low SA score values. Such expansion is relevant to chemical space beyond the rule of five.^86^ Although natural products are an important source of such compounds, structure-based design also contributes to drug discovery in this space.^87^ As shown in Figure S6, SF-GA generated molecules across a wide range of molecular weights, including molecules beyond the rule of five. Notably, SA score values remained relatively low at higher molecular weights.

### 3.2 Effect of Synergy-Guided Selection

In this work, mutation is guided by synergy groups derived from the MACCS-XGBoost model, while generated molecules are evaluated using the ensemble model. Since different models are used for guidance and evaluation, we compared predictions from the MACCS-XGBoost and ensemble models for molecules generated by SF-GA (selection off). The Pearson’s correlation coefficient between their predictions was 0.885 (Figure S3), indicating strong agreement in the predicted probabilities. This result suggests that MACCS-XGBoost-derived synergy groups can guide mutation under ensemble evaluation. In addition, we compared the evaluation functions using the MACCS-XGBoost and ensemble models (Table S5).

To evaluate whether the performance gain is attributable to bit co-occurrence patterns rather than SHAP-derived importance alone, two control models were constructed. Only the fragment selection step was modified, while all other components of SF-GA were kept unchanged. F-GA selects replacement fragments uniformly at random from the same BRICS-compatible fragment library, subject to the same attachment-point constraints and acceptance criterion. Single-bit-GA ranks candidate fragments according to the sum of the mean SHAP values of their active bits, using the same SHAP-derived importance scores as SF-GA but without considering parent-fragment identity or these patterns. All three methods employ the same BRICS-constrained replacement framework, fragment library, and acceptance criterion. Thus, F-GA and Single-bit-GA serve as controls for unguided and importance-only fragment selection, respectively. These models allow the contribution of bit co-occurrence patterns to be evaluated separately.

The same initial pool of 100 randomly selected P-gp substrates was used for SF-GA, Single-bit-GA, and F-GA in each independent run. Figure 3 shows the distribution of *p̂*_nonsubs_ among elite molecules across generations. To compare the optimization rates among the models, the AUCs were compared using paired *t*-tests. SF-GA showed a significantly higher AUC than Single-bit-GA (*p*  < 0.001) and F-GA (*p*  < 0.001), indicating faster optimization.

**Figure 3:**
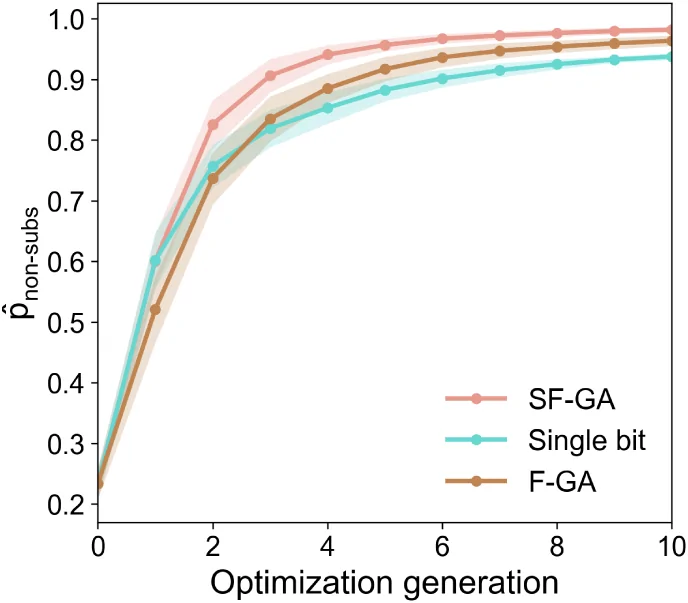
Generation-dependent distributions of *p̂*_nonsubs_. Shaded regions represent standard deviations across molecules.

To examine the structural changes associated with synergy-guided mutation, five representative molecules were tracked. For each molecule, if an offspring exhibited a lower *p̂*_nonsubs_ value than its parent, the parent was retained. Each experiment was repeated 30 times, and the mean *p̂*_nonsubs_ value was calculated for each generation. The trajectories of the five representative molecules are shown in Figure 4. SF-GA generally increased *p̂*_nonsubs_ more rapidly than the other ablation models. After the 10th generation, SF-GA consistently yielded the highest *p̂*_nonsubs_ values among the tracked molecules.

**Figure 4:**
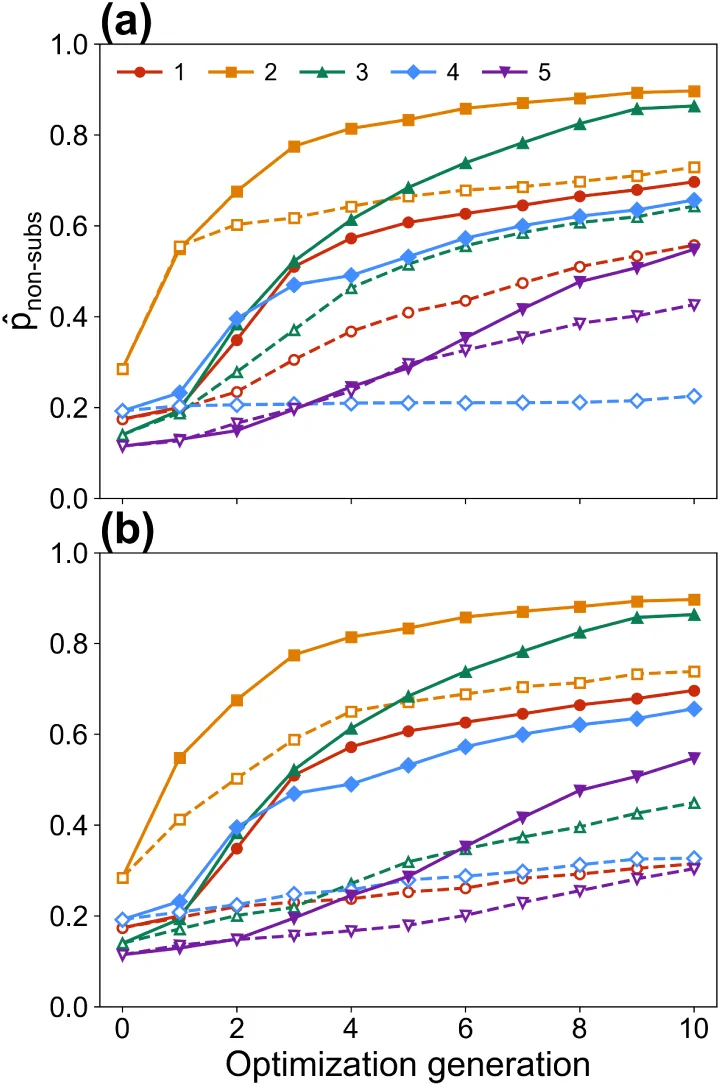
Tracking of five molecules: (a) using SF-GA (solid line) and Single bit-GA (dashed line), (b) SF-GA (solid line) and F-GA (dashed line). See Supporting Information, Table S6 for details.

SF-GA, Single-bit-GA, and F-GA share the same BRICS-constrained fragment replacement framework, fragment library, attachment-point constraints, and acceptance rule. The only difference among the three methods is the criterion used to select replacement fragments. SF-GA selects fragments based on bit co-occurrence patterns, Single-bit-GA ranks fragments according to their mean SHAP values, and F-GA selects fragments randomly.

In this setting, differences in the rate of improvement reflect differences in exploration efficiency rather than differences in the underlying search space. By preferentially replacing fragments with negative synergy using candidates with positive synergy, SF-GA increases *p̂*_nonsubs_ more rapidly than Single-bit-GA. As a result, SF-GA reaches higher *p̂*_nonsubs_ values earlier across generations (Figure 4). While F-GA shows that random fragment replacement can occasionally improve the target property, the rapid improvement observed with Single-bit-GA for several molecules suggests that individual bit importance can contribute to effective fragment selection. Nevertheless, SF-GA consistently reaches higher final probabilities than Single-bit-GA for all tested molecules, suggesting that bit co-occurrence captures structural context not captured by individual bit importance alone.

In addition to improving convergence, SF-GA retains information on the fragments removed and inserted during each accepted mutation, together with their synergy scores. This enables fragment-level analysis of changes in *p̂*_nonsubs_.

### 3.3 Effect of Fragment Number on Optimization Efficiency

At each mutation step, SF-GA replaces a single fragment, and its optimization efficiency depends on the number of fragments in the molecule. The combinatorial space can be approximated by $\Omega =\overset{k}{\underset{i=1}{\prod }}n_{i}$ , where *k* is the number of fragments and *n*_*i*_ denotes the number of feasible fragment candidates at position *i* subject to the attachment-point and BRICS isotope constraints. As *k* increases, random replacement becomes inefficient for sampling favorable fragment combinations.

Hence, changes in *p̂*_nonsubs_ were tracked for 10 randomly selected molecules with three, five, and seven fragments. Each molecule was optimized for 10 generations, with each experiment repeated 10 times using SF-GA, Single-bit-GA, and F-GA (Figure 5).

**Figure 5:**
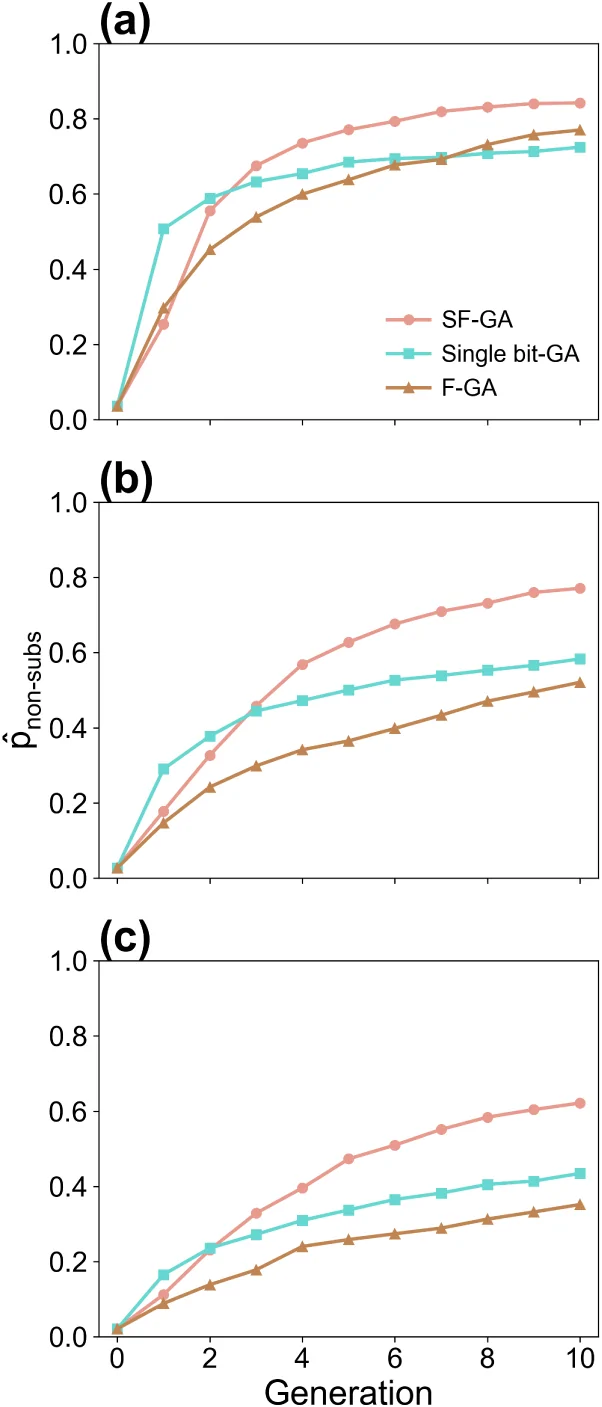
Generation dependence of *p̂*_nonsubs_ for individual molecules evolved by SF-GA, Single-bit-GA, and F-GA, grouped by fragment count: (a) 3 fragments, (b) 5 fragments, and (c) 7 fragments. Solid lines represent mean values. See Table S7 in the Supporting Information for details.

For SF-GA, molecules with more fragments generally required more generations to achieve improvement, indicating a slower convergence rate. Nevertheless, the mean *p̂*_nonsubs_ exceeded the decision threshold within 10 generations. In contrast, Single-bit-GA and F-GA showed a stronger dependence on the number of fragments. Molecules containing three fragments reached the decision threshold within 10 generations on average, whereas those containing seven fragments showed only limited improvement in *p̂*_nonsubs_ and did not reach the threshold within 10 generations. These results suggest that synergy-guided mutation improves optimization efficiency and reduces the dependence on fragment number observed with Single-bit-GA and F-GA.

### 3.4 Molecular Editing Using SF-GA

In drug discovery, structure–activity relationship (SAR) analysis typically involves systematic molecular modifications to understand changes in biological activity and associated ADMET properties. To reflect molecular optimization practice, we evaluated SF-GA using peripheral editing, which preserves an anchor scaffold and is characterized using Bemis–Murcko scaffolds,^88^ a standard representation of molecular core structures obtained by removing side chains.

SF-GA was designed for cases where an anchor scaffold plays an important role in determining activity and binding pose in the lead optimization stage of drug development. Therefore, it enables scaffold-preserving molecular editing, in which modifications are restricted to peripheral fragments while the central scaffold is retained. To demonstrate this, we mimicked late-stage lead optimization by modifying functional groups while keeping the core scaffold fixed.

For each molecule, the Bemis-Murcko scaffold was identified as the anchor scaffold. Mutations were restricted to peripheral fragments attached to the scaffold. Under the scaffold constraint, all three substrate molecules exceeded a *p̂*_nonsubs_ value of 0.7 after the first generation (Figure 6). Editing the peripheral substructures substantially increased *p̂*_nonsubs_, suggesting compatibility between the selected fragments and the scaffold. To assess synthetic feasibility, retrosynthetic routes for these molecules are shown in Figure S7. The same framework was applied to a P-gp nonsubstrate molecule, resulting in an increase in P-gp substrate probability (Figure S9a). In addition, SF-GA can be used for scaffold optimization under a similarity constraint. Generated molecules are required to remain similar to the starting compound while altering the Bemis-Murcko scaffold.^88,89^ This enables optimization of individual molecules rather than generation of entirely new molecules.

**Figure 6:**
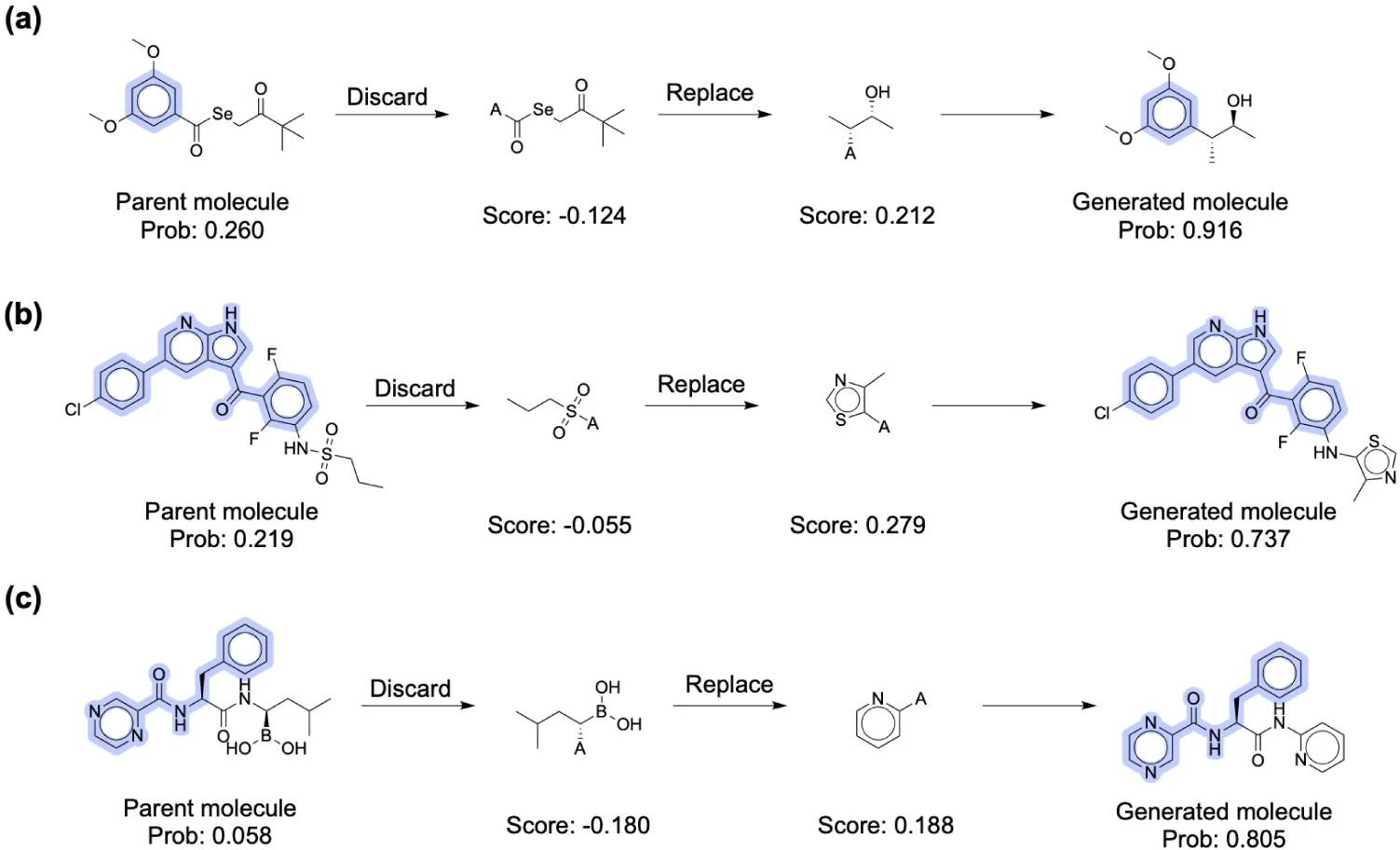
Simulation of score-driven peripheral editing using SF-GA for three P-gp substrate molecules with synergy scores. (a, b, c) Three representative peripheral optimization examples under the fixed scaffold constraint. The scaffold of each molecule is highlighted in purple.

Three additional conditions were applied to the standard SF-GA mutation step. First, fragment replacement was guided by structural similarity. For each candidate satisfying the positive-synergy criterion, the maximum common substructure (MCS) with the discarded fragment was identified, and the number of atoms not included in the MCS was used to quantify structural difference. These values were min-max normalized and converted into sampling probabilities. Structurally similar fragments were assigned higher probabilities while retaining stochastic sampling. Second, each generated molecule was required to have a Tanimoto similarity of at least 0.5 to the starting molecule. The starting molecule was used as the similarity reference to limit cumulative structural drift over successive generations. Third, fragment replacement was confined to the Bemis–Murcko scaffold of each parent molecule. Generated molecules were examined for structural changes relative to the parent molecule.

SF-GA increased the predicted probability of P-gp nonsubstrate activity through similarity-guided fragment replacement. As shown in Figure 7a, the optimized molecule retained high structural similarity to the original molecule (Tanimoto similarity = 0.721) while showing a substantial increase in *p̂*_nonsubs_. Figure 7b shows that a lower structural similarity (0.576) was associated with a greater increase in the predicted probability. To consider synthetic feasibility, retrosynthetic routes for the resulting molecules were generated using ASKCOS^90^ and are shown in Figure S8. The same framework increased the predicted P-gp substrate probability of a P-gp nonsubstrate molecule through synergy-guided mutation (Figure S9 c).

**Figure 7:**
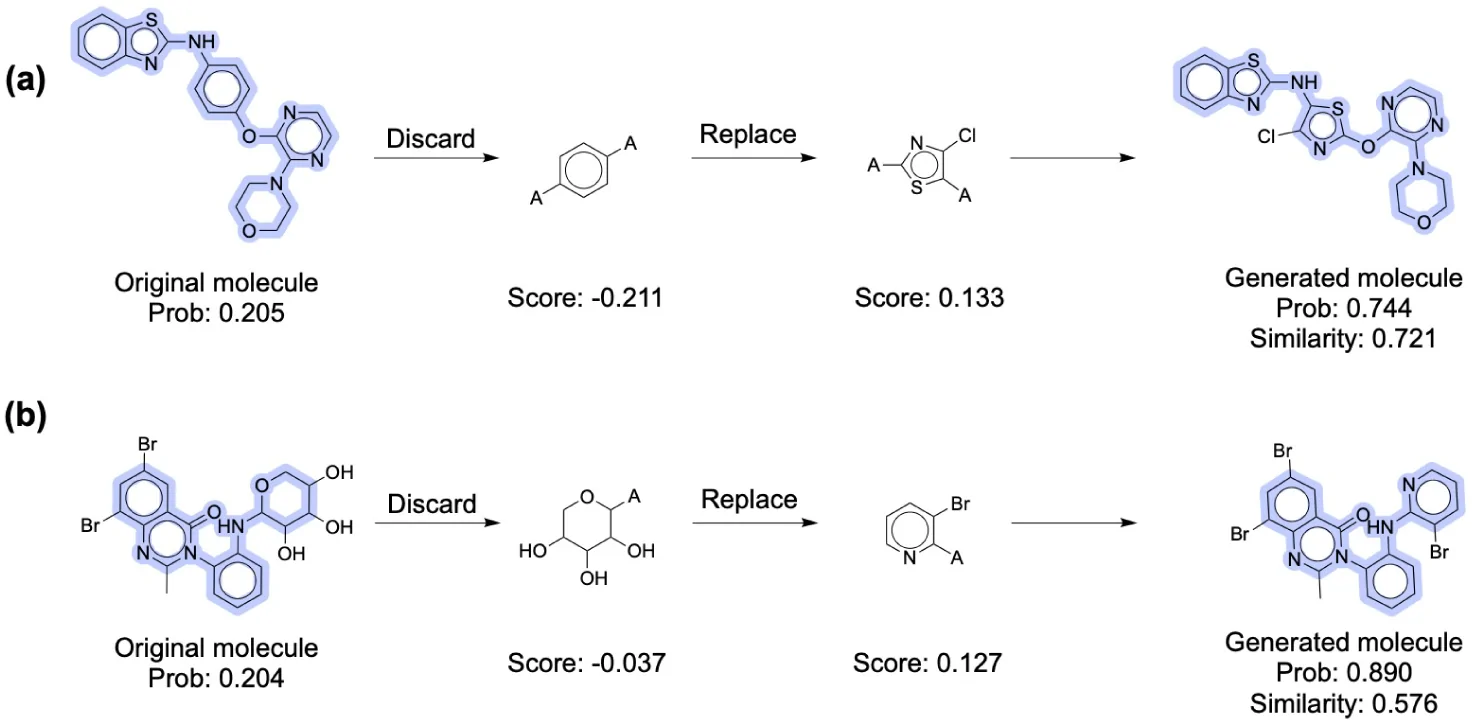
High-similarity editing simulations using SF-GA on two P-gp substrate molecules with synergy scores. (a, b) Two representative scaffold-optimization examples under the similarity constraint. The scaffold of each molecule is highlighted in purple.

In lead optimization, exploring peripheral substitutions on a common scaffold and optimizing related molecules are common strategies for expanding SAR coverage. A key challenge is generating molecules with the desired property while retaining the core scaffold. SF-GA addresses this challenge by selecting replacement fragments based on their synergy with the retained scaffold and neighboring fragments. Repeated scaffold-preserving mutations generate diverse combinations of peripheral fragments. The synergy score for each replacement also provides a fragment-level interpretation of the resulting structural changes. This enables the selection of structurally diverse candidates based on synthetic accessibility and property balance, supporting in silico molecular exploration in late-stage drug development.

### 3.5 Molecular Docking Simulation

Docking simulations were performed for molecules generated by SF-GA. To examine the relationship between *p̂*_nonsubs_ and docking scores, 10,156 molecules generated over five generations via SF-GA (selection off) were analyzed. Before docking, molecules were filtered using the NIH,^75^ Brenk,^74^ and PAINS^73^ filters to remove compounds likely to produce assay artifacts or exhibit unfavorable drug-like properties. A total of 1,570 molecules remained after filtering. The filtered molecules were converted to initial 3D geometries using OpenBabel.^91^ Docking simulations were performed using AutoDock Vina.^92,93^ Two 3D structures of human P-gp (PDB ID: 6QEX^94^ and PDB ID: 7O9W^95^) were obtained from the Protein Data Bank, and a cubic grid box with a 22.5 Å side length was centered on the cocrystallized ligands to define potential binding sites.

To benchmark docking scores, four reference molecules with different P-gp interaction types were included. Paclitaxel, a well-characterized P-gp substrate, was included as a positive control.^96^ Encequidar, a potent, nonabsorbable P-gp inhibitor, served as a nonsubstrate reference despite its high affinity for the substrate-binding domain.^97^ Carbamazepine and 5-fluorouracil were included as negative controls because they show minimal interaction with P-gp.^98,99^

Following the results shown in Figure 8, the relationship between the *p̂*_nonsubs_ and the computed docking scores for the generated molecules showed a general upward trend, particularly when *p̂*_nonsubs_ exceeded the 0.540 threshold. As *p̂*_nonsubs_ increased beyond 0.540, docking scores generally tended to rise. Moreover, in Figure S10, P-gp nonsubstrates were observed more than substrates during the weak binding affinity interval. This trend suggests that molecules generated as predicted P-gp nonsubstrates by the SF-GA model also exhibit weak binding affinities toward P-gp in molecular docking simulations.

**Figure 8:**
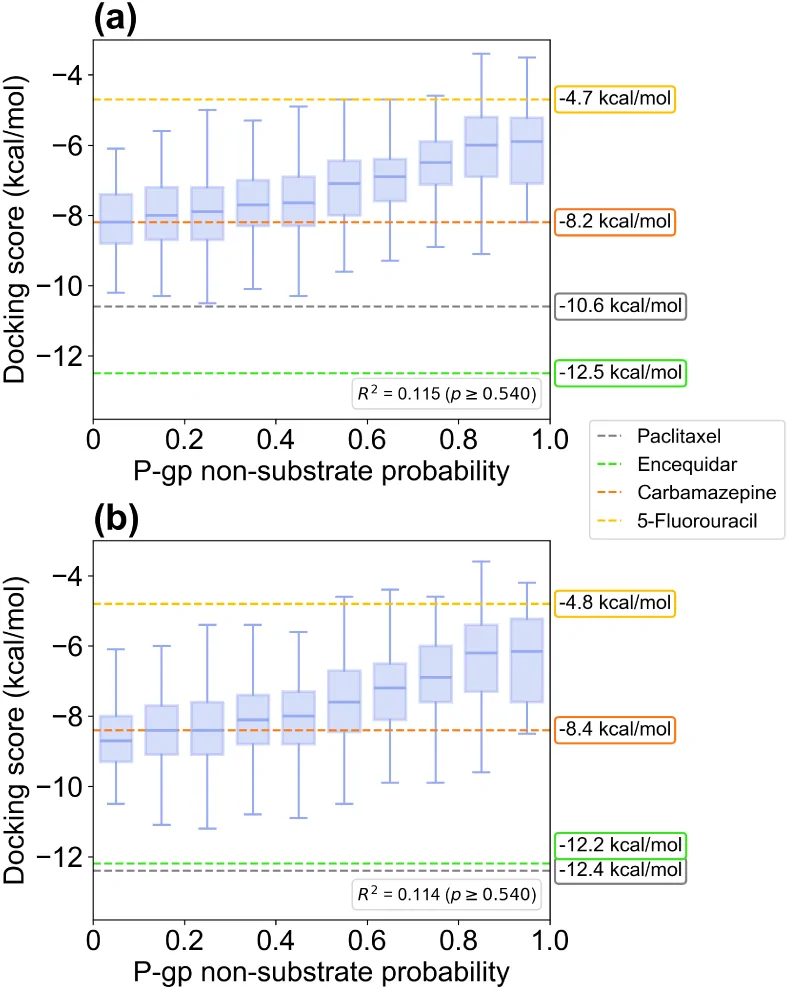
Box plots of docking scores for ligands generated by SF-GA in human P-gp, grouped by predicted nonsubstrate probabilities in 0.1 increments. (a) PDB ID: 6QEX. (b) PDB ID: 7O9W.

Furthermore, ligand 4, one of the high *p̂*_nonsubs_ molecules generated by SF-GA, was compared with paclitaxel to investigate the structural basis of weak P-gp interactions. Ligand 4 exhibited a *p̂*_nonsubs_ of 0.979 and docking scores of −5.1 kcal mol^–1^ and −5.2 kcal mol^–1^ in PDB ID: 6QEX and 7O9W structures (full docking results are provided in the SF-GA docking scores file as Supporting Information). Key neighboring residues, including Gln725, Gln347, and Gln990, previously reported to form hydrogen bonds with paclitaxel,^96^ were consistently observed in Figure 9c. In contrast, ligand 4 formed no hydrogen bonds despite occupying a similar binding site (Figure 9b,d), consistent with its predicted weak P-gp binding. Similarly, ligand 4 bound to 7O9W occupied a binding region comparable to that of encequidar bound to 7O9W (Figure 10) and was located near several common neighboring residues, although it exhibited weaker predicted binding interactions. Additionally, docking poses of ligand 1, 2, and 3 with 6QEX and 7O9W are represented in Figure S12 and Figure S13.

**Figure 9:**
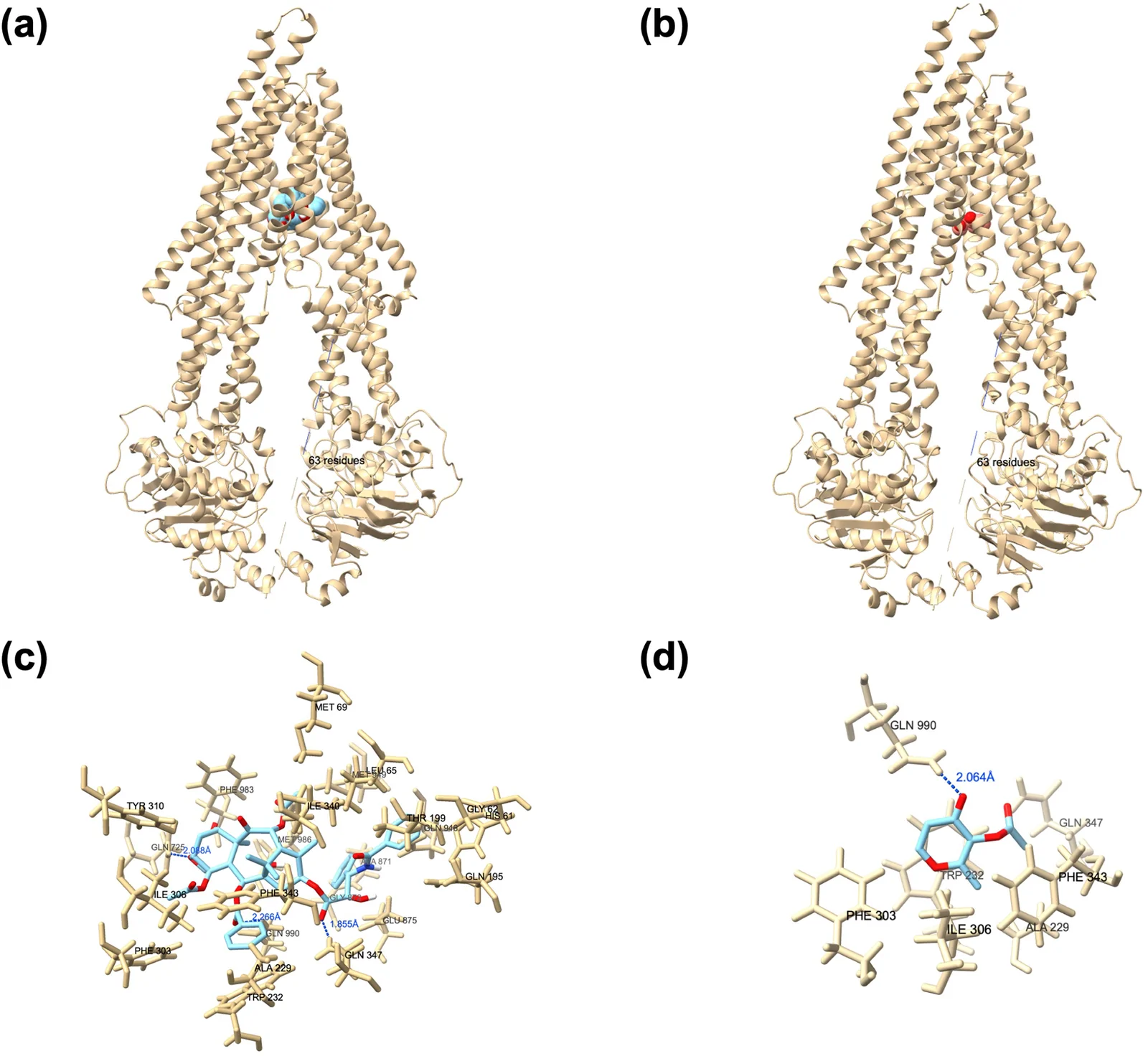
Comparative molecular docking simulations of paclitaxel and a ligand bound to human P-gp (PDB ID: 6QEX). (a, c) Binding modes of paclitaxel and (b, d) ligand 4 within the internal cavity of P-gp. Detailed views (c, d) highlight residues within 3.5 Å of the ligands and specific hydrogen-bond networks (sky blue).

**Figure 10:**
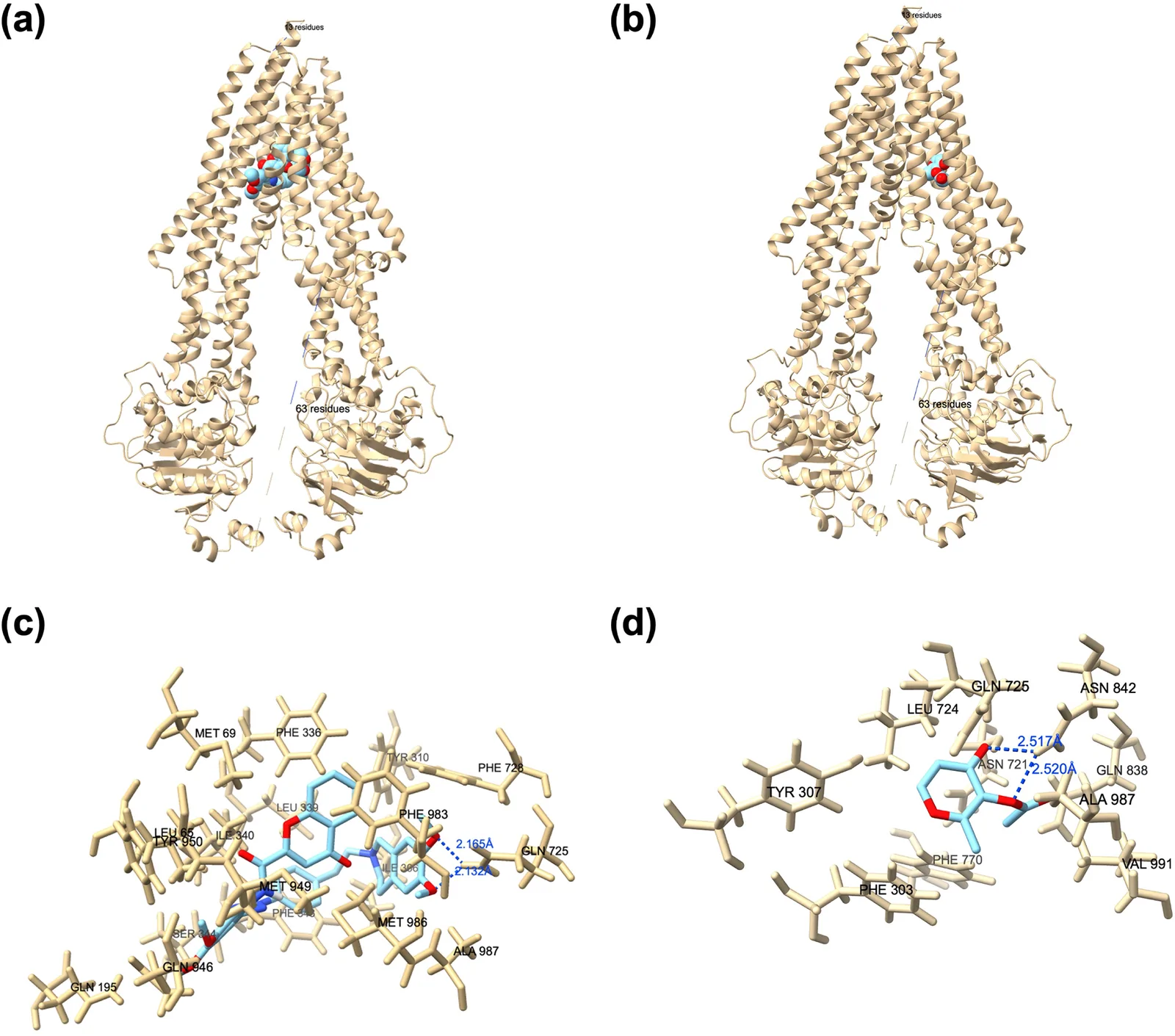
Comparison of docking poses of encequidar and ligand 4 in P-gp (PDB ID: 7O9W). (a, c) Encequidar and (b, d) ligand 4 bound within the P-gp binding pocket. Ligands are shown in magenta. Panels (c) and (d) depict residues within 3.5 Å of the ligands and hydrogen-bonding interactions.

A subset of the generated molecules that predicted nonsubstrate still exhibited relatively low docking scores, indicating stronger binding affinities. This is consistent with the behavior of certain nonsubstrates, such as encequidar, which can bind strongly despite not being transported. Overall, these findings highlight the diverse interaction profiles within the nonsubstrate chemical space and demonstrate that SF-GA can efficiently explore this space for molecules with weak P-gp interactions.

## 4 Conclusion

We present SF-GA, a synergy-guided fragment genetic algorithm for converting P-gp substrates into predicted nonsubstrates via fragment-level molecular editing. By incorporating SHAP-derived synergistic group analysis into the mutation stage, SF-GA replaces fragments using synergy scores, providing a chemically interpretable basis for structural modifications.

Under a fixed number of function evaluations, SF-GA achieved optimization performance comparable to GB-GA, REINVENT, and FragGT while generating molecules with relatively low SA scores. Ablation studies against F-GA and Single bit-GA indicate that bit co-occurrence provides additional guidance for increasing the predicted nonsubstrate probability, with this trend maintained as the number of fragments increases. By recording each fragment replacement together with its synergy score, SF-GA further links changes in *p̂*_nonsubs_ to specific substructural modifications under a fixed scaffold. Docking against two human P-gp structures showed weaker predicted binding for molecules with higher *p̂*_nonsubs_. These results provide structural characterization of the generated molecules but do not directly establish nonsubstrate behavior.

Overall, this study introduces a computational framework for optimizing the predicted *p̂*_nonsubs_. Because the generated molecules are hypothetical and *p̂*_nonsubs_ is estimated using an ensemble model, the observed trends should be interpreted as model-based predictions rather than experimentally validated results. SF-GA uses synergy groups derived from SHAP values of substructural features. Hence, its application assumes that the target property can be represented by explicit substructural contributions and may be less suitable for properties dominated by whole-molecule physicochemical descriptors. The resulting guidance also depends on the reliability of the SHAP values used to derive the synergy groups, particularly when features are highly correlated. Data set size and class imbalance should also be considered when applying SF-GA to new molecular properties. SF-GA can also be extended to molecular optimization problems where substructural contributions are relevant.

## Supplementary Material





## Data Availability

The source code for the SF-GA related to interconversion between P-gp substrate and P-gp nonsubstrate is available on GitHub at https://github.com/hwk-grp/SF-GA.

## Notes

Complete molecular docking results for 1,570 SF-GA-generated molecules against 6QEX and 7O9W (CSV)
Physicochemical properties of the P-gp data set; benchmarking of predictive models and molecular representations; threshold analysis of the SF-GA mutation step; comparison of chemical space among generated molecules; additional ablation analyses and details of representative molecules; synthetic accessibility and retrosynthetic analyses; molecular editing examples; docking score analyses and binding modes in P-gp structures 6QEX and 7O9W (PDF)
