# Supplementary material for: Synergy-Guided Molecular Redesign of Predicted P‑Glycoprotein Substrates toward Synthetically Feasible Non-Substrates
Source: J Chem Inf Model. 2026 Oct 19;66(21):14174–87. doi: 10.1021/acs.jcim.6c01938 (PMC13753732; doi:10.1021/acs.jcim.6c01938)
Supplement: Supplementary file 1 [file ci6c01938_si_002.pdf]

Supporting Information:
Synergy-Guided Molecular Redesign of Predicted P-glycoprotein Substrates toward Synthetically Feasible Non-Substrates

Soyeon Jeon,[†] Sung Wook Moon,[†] and Hyun Woo Kim[*,†,‡]

†*Department of Chemistry, Gwangju Institute of Science and Technology, Gwangju, 61005, Republic of Korea*

‡*Center for Quantum Technology, Korea Institute of Science and Technology, Seoul 02792, Republic of Korea*

E-mail: hwk@gist.ac.kr

Table S1: Comparison of predictive performance among machine learning models. Extreme gradient boosting (XGBoost), random forest (RF), support vector machine (SVM), and deep neural network (DNN) models were trained using MACCS key fingerprints as input features, whereas graph convolutional network (GCN) and AttentiveFP models were trained using molecular graph representations. The dataset was first split into training and test sets at a 9:1 ratio. For models requiring a validation set, the training set was further split into training and validation sets at a 9:1 ratio. Model performance was evaluated using ROC-AUC, accuracy, F1 score, precision, recall, and Matthews correlation coefficient (MCC). All values are reported as mean ± standard deviation across five independent random seeds.

Model	ROC-AUC	Accuracy	F1 score	Precision	Recall	MCC
**XGBoost**	**0.883 ± 0.017**	**0.805 ± 0.018**	**0.819 ± 0.018**	**0.807 ± 0.020**	0.832 ± 0.034	**0.608 ± 0.035**
RF	0.876 ± 0.020	0.804 ± 0.024	0.818 ± 0.025	**0.807 ± 0.019**	0.830 ± 0.042	0.607 ± 0.049
SVM	0.868 ± 0.008	0.795 ± 0.010	0.812 ± 0.012	0.793 ± 0.014	**0.834 ± 0.031**	0.590 ± 0.022
DNN	0.853 ± 0.019	0.771 ± 0.025	0.792 ± 0.022	0.766 ± 0.029	0.821 ± 0.042	0.541 ± 0.049
GCN	0.798 ± 0.028	0.714 ± 0.018	0.754 ± 0.026	0.693 ± 0.015	0.832 ± 0.072	0.433 ± 0.041
AttentiveFP	0.877 ± 0.015	0.793 ± 0.030	0.814 ± 0.024	0.784 ± 0.041	0.847 ± 0.035	0.587 ± 0.059

Table S2: Comparison of predictive performance using different molecular fingerprints. All fingerprints were used as input features for XGBoost. The dataset was first split into training and test sets at a 9:1 ratio. Model performance was evaluated using ROC-AUC, accuracy, F1 score, precision, recall, and Matthews correlation coefficient (MCC). All values are reported as mean ± standard deviation across five independent random seeds.

Fingerprint	ROC-AUC	Accuracy	F1 score	Precision	Recall	MCC
**MACCS**	**0.883 ± 0.017**	**0.805 ± 0.018**	0.819 ± 0.018	**0.807 ± 0.020**	0.832 ± 0.034	**0.608 ± 0.035**
ECFP4	0.878 ± 0.010	0.787 ± 0.007	0.808 ± 0.006	0.778 ± 0.009	0.840 ± 0.012	0.573 ± 0.013
RDKit FP	0.877 ± 0.003	0.786 ± 0.007	0.811 ± 0.006	0.764 ± 0.007	0.836 ± 0.007	0.572 ± 0.014
PubChem FP	0.878 ± 0.008	0.801 ± 0.012	**0.826 ± 0.010**	0.772 ± 0.013	**0.887 ± 0.009**	0.604 ± 0.024

Table S3: Candidate counts for different thresholds in 100 molecules.

Threshold	Discard candidates	Replace candidates
0	4.81 ± 2.47	4220.19 ± 2999.28
μ	2.24 ± 1.46	1824.22 ± 1341.40
$\mu \pm \sigma$	1.19 ± 0.72	700.15 ± 524.49
$\mu \pm 2\sigma$	0.18 ± 0.46	170.33 ± 121.68

Table S4: The ensemble model combined predictions from three XGBoost models trained using different molecular representations, including MACCS, ECFP4, and RDKit descriptors. Here, for the RDKit-based model, both fragment-level and molecular-level descriptors were used. The decision threshold of the ensemble model was either fixed at 0.5 or determined using the Youden J statistic. Performance was evaluated using ROC-AUC, accuracy, F1 score, precision, recall, and Matthews correlation coefficient (MCC). All values are reported as mean ± standard deviation across five independent random seeds.

Model	ROC-AUC	Accuracy	F1 score	Precision	Recall	MCC
XGBoost (MACCS)	0.883 ± 0.017	0.805 ± 0.018	0.819 ± 0.018	0.807 ± 0.020	0.832 ± 0.034	0.608 ± 0.035
XGBoost (ECFP4)	0.878 ± 0.010	0.787 ± 0.007	0.808 ± 0.006	0.778 ± 0.009	0.840 ± 0.012	0.573 ± 0.013
XGBoost (RDKit descriptor)	0.900 ± 0.006	0.812 ± 0.012	0.831 ± 0.010	0.795 ± 0.014	0.871 ± 0.008	0.623 ± 0.024
AttentiveFP	0.877 ± 0.015	0.793 ± 0.030	0.814 ± 0.024	0.784 ± 0.041	0.847 ± 0.035	0.587 ± 0.059
Ensemble	0.909 ± 0.002	0.806 ± 0.011	0.825 ± 0.009	0.793 ± 0.015	0.860 ± 0.013	0.611 ± 0.021
Ensemble (Youden J)	0.909 ± 0.002	0.808 ± 0.014	0.823 ± 0.011	0.807 ± 0.020	0.840 ± 0.010	0.614 ± 0.027

Table S5: Performance of SF-GA with different evaluation functions during the selection step using the MACCS-XGBoost and ensemble models. AUC top-50 denotes the area under the curve of the average predicted non-substrate probability ($\hat{p}_{\text{non-subs}}$) of the top 50 molecules as a function of the number of function evaluations, normalized to a budget of 5,000 evaluations. All remaining metrics were computed for the top 50 molecules at the end of the evaluation budget. Values are reported as mean ± standard deviation over 10 independent runs.

Metric	Ensemble	MACCS-XGBoost
AUC top-50	0.948 ± 0.004	0.960 ± 0.003
$\hat{p}_{\mathrm{non-subs}}$	0.992 ± 0.001	0.997 ± 0.000
QED	0.431 ± 0.044	0.362 ± 0.106
SAscore	2.962 ± 0.159	3.321 ± 0.321
SYBA	40.9 ± 10.4	42.6 ± 11.9
RAscore	0.932 ± 0.037	0.804 ± 0.076
MW	359.3 ± 38.9	429.5 ± 120.5
Diversity	0.744 ± 0.041	0.790 ± 0.041
Novelty	0.721 ± 0.016	0.753 ± 0.014

Table S6: Representative molecules, corresponding fragment counts, and predicted probabilities of P-gp non-substrate classification.

No.	SMILES	Fragment count	Initial prob.	Final prob.		
				F-GA	Single bit-GA	SF-GA
1	Cc1cc([C@@]2(C)C[NH2+][C@@H](C)CN2c2cccc3c2OCC[C@@H]3[NH2+]S(=O)(=O)CCC(=O)N2C(=O)[C@H]3CCC[NH+]3[C@@H]2c2nnc3n2N[C@H]([C@H]2C=C(c4ccc(-c5ccc6c(c5O)C(=O)c5ccccc5C6=O)cc4)N=N2)S3)c2c(SC3=NC(=O)[C@H](C#N)C(C)(C)[C@@H]3C#N)noc2n1	8	0.174	0.335	0.525	0.686
2	N#CC1(CCN(CC1)Cc(cc2c3CN(C2=O)[C@H]4CCCC[C@@H]4O)c5c3cccc5)c6nccnc6	5	0.285	0.714	0.728	0.886
3	Cc1c([C@@H]2COc3ccccc3O2)oc2ccc3c(c12)[C@@H](O[C@@H](C)C1=CC[NH+](S(=O)(=O)CCC(=O)O/N=C(/N)c2ccccc2)CC1)CC(C)(C)O3	5	0.140	0.499	0.622	0.869
4	Cc1c([C@H](C)C(=O)[C@@H](C#N)c2cc(Br)cc(/C=N/Nc3ccc([N+](=O)[O-])cn3)c2O)sc2ccc([C@@H]3NC(=O)NC4=C3C(=O)N(C(=O)CCS(=O)(=O)N3C[C@H](C)[NH2+]C[C@H]3C(=O)N/N=C\c3cc(N/C([S-])=C(/C#N)c5nc6ccccc6s5)c(O)c([N+](=O)[O-])c3)C4)cc12	8	0.192	0.379	0.215	0.627
5	Cc1ncc(S(C)(=O)=O)c([C@@H]2[C@H](c3cccc4c(C5=CCN(c6ccc7c(c6)[C@@]6(CC7)C[NH2+]C[C@H](N7CCC8(CC7)CN(C)C(=O)O8)O6)CC5)c[nH]c34)CCN2C(=O)CCS(=O)(=O)N2CCN(C)c3ccccc3C2)n1	7	0.115	0.306	0.445	0.558

Table S7: Mean ± standard deviation of the predicted P-gp non-substrate probability at each generation for different fragment counts, calculated from 10 independent runs for each of 10 random seeds.

Fragment count	Generation	SF-GA	Single bit-GA	F-GA
3	0	0.035 ± 0.024	0.035 ± 0.024	0.035 ± 0.024
	1	0.254 ± 0.214	0.507 ± 0.248	0.297 ± 0.312
	2	0.555 ± 0.287	0.589 ± 0.213	0.452 ± 0.301
	3	0.675 ± 0.252	0.633 ± 0.185	0.539 ± 0.274
	4	0.736 ± 0.223	0.654 ± 0.171	0.599 ± 0.255
	5	0.771 ± 0.201	0.685 ± 0.145	0.638 ± 0.242
	6	0.793 ± 0.187	0.694 ± 0.141	0.677 ± 0.217
	7	0.819 ± 0.157	0.698 ± 0.140	0.692 ± 0.214
	8	0.831 ± 0.147	0.708 ± 0.135	0.731 ± 0.192
	9	0.841 ± 0.144	0.713 ± 0.133	0.758 ± 0.177
	10	0.842 ± 0.144	0.724 ± 0.127	0.770 ± 0.166
5	0	0.027 ± 0.017	0.027 ± 0.017	0.027 ± 0.017
	1	0.178 ± 0.145	0.290 ± 0.270	0.147 ± 0.184
	2	0.327 ± 0.215	0.378 ± 0.250	0.242 ± 0.224
	3	0.458 ± 0.237	0.444 ± 0.226	0.298 ± 0.225
	4	0.569 ± 0.263	0.472 ± 0.209	0.342 ± 0.235
	5	0.627 ± 0.251	0.500 ± 0.206	0.365 ± 0.228
	6	0.676 ± 0.228	0.527 ± 0.189	0.398 ± 0.220
	7	0.710 ± 0.194	0.539 ± 0.183	0.433 ± 0.215
	8	0.732 ± 0.178	0.553 ± 0.173	0.471 ± 0.231
	9	0.760 ± 0.168	0.566 ± 0.156	0.495 ± 0.236
	10	0.771 ± 0.163	0.583 ± 0.153	0.521 ± 0.237
7	0	0.021 ± 0.010	0.021 ± 0.010	0.021 ± 0.010
	1	0.112 ± 0.089	0.165 ± 0.192	0.089 ± 0.086
	2	0.231 ± 0.167	0.236 ± 0.186	0.138 ± 0.116
	3	0.329 ± 0.204	0.272 ± 0.187	0.178 ± 0.140
	4	0.396 ± 0.230	0.310 ± 0.184	0.240 ± 0.166
	5	0.473 ± 0.235	0.337 ± 0.174	0.259 ± 0.177
	6	0.510 ± 0.221	0.365 ± 0.180	0.274 ± 0.176
	7	0.551 ± 0.212	0.382 ± 0.185	0.289 ± 0.177
	8	0.584 ± 0.202	0.405 ± 0.181	0.313 ± 0.176
	9	0.604 ± 0.192	0.414 ± 0.178	0.332 ± 0.174
	10	0.622 ± 0.187	0.435 ± 0.186	0.352 ± 0.179

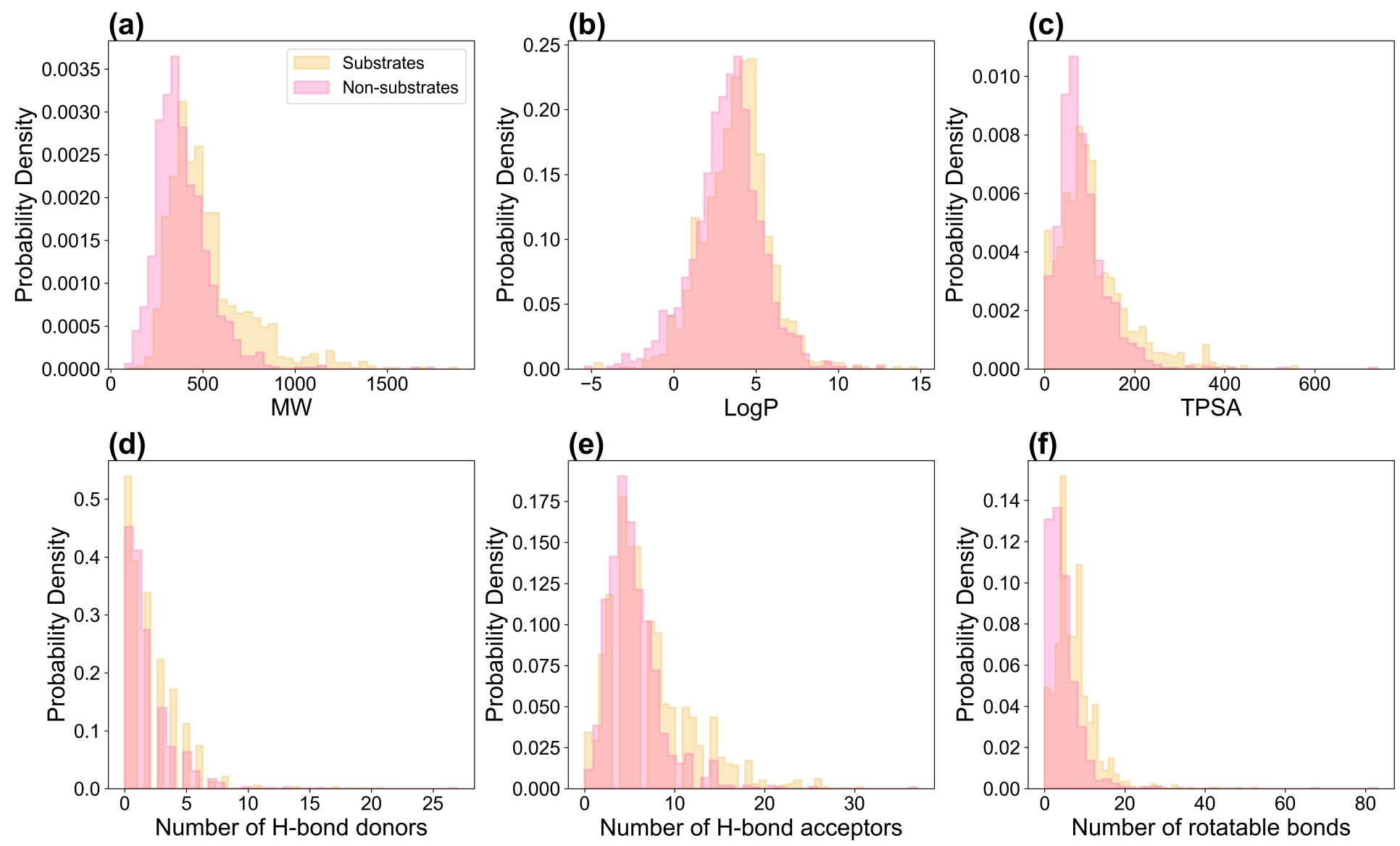

Figure S1: Distributions of Lipinski-related physicochemical properties for in the dataset: (a) molecular weight, (b) LogP, (c) topological polar surface area (TPSA), (d) number of hydrogen-bond donors, (e) number of hydrogen-bond acceptors, and (f) number of rotatable bonds. Each P-gp substrate class is shown as different colors.

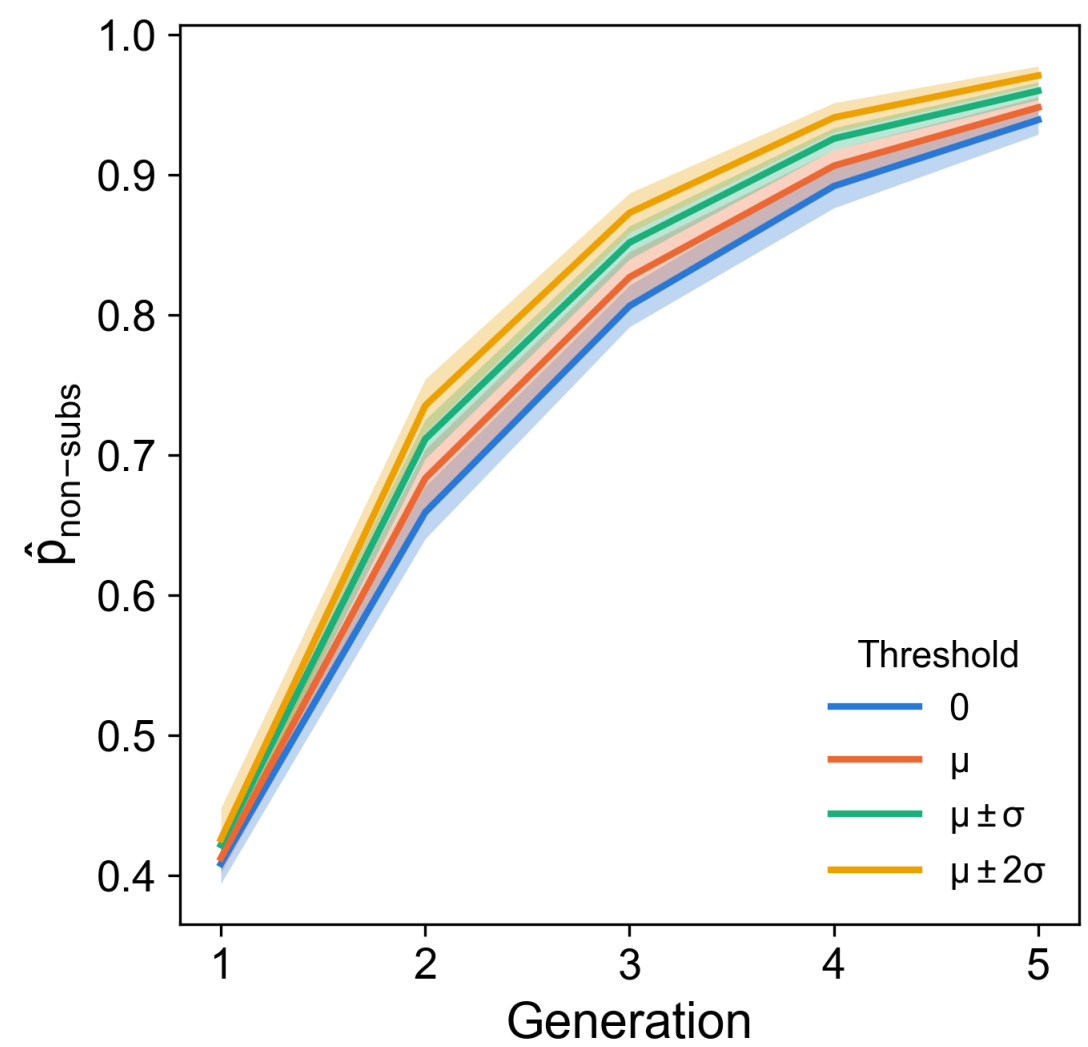

Figure S2: Comparison of optimization efficiency while changing the threshold of synergy score in mutation step.

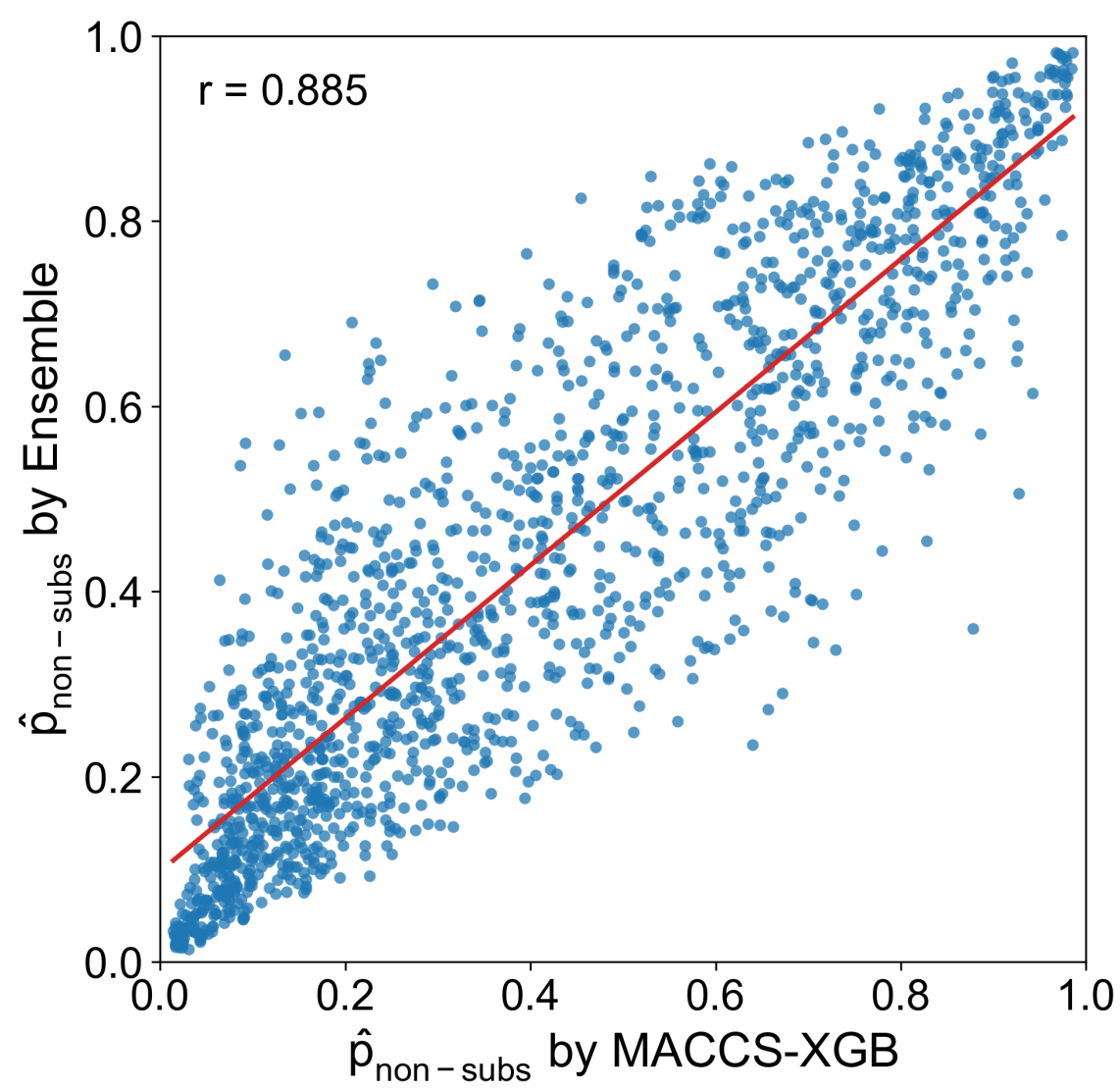

Figure S3: Correlation between predicted non-substrate probabilities from the MACCS-XGBoost and ensemble models. Pearson's correlation coefficient and the linear regression line (red) are shown.

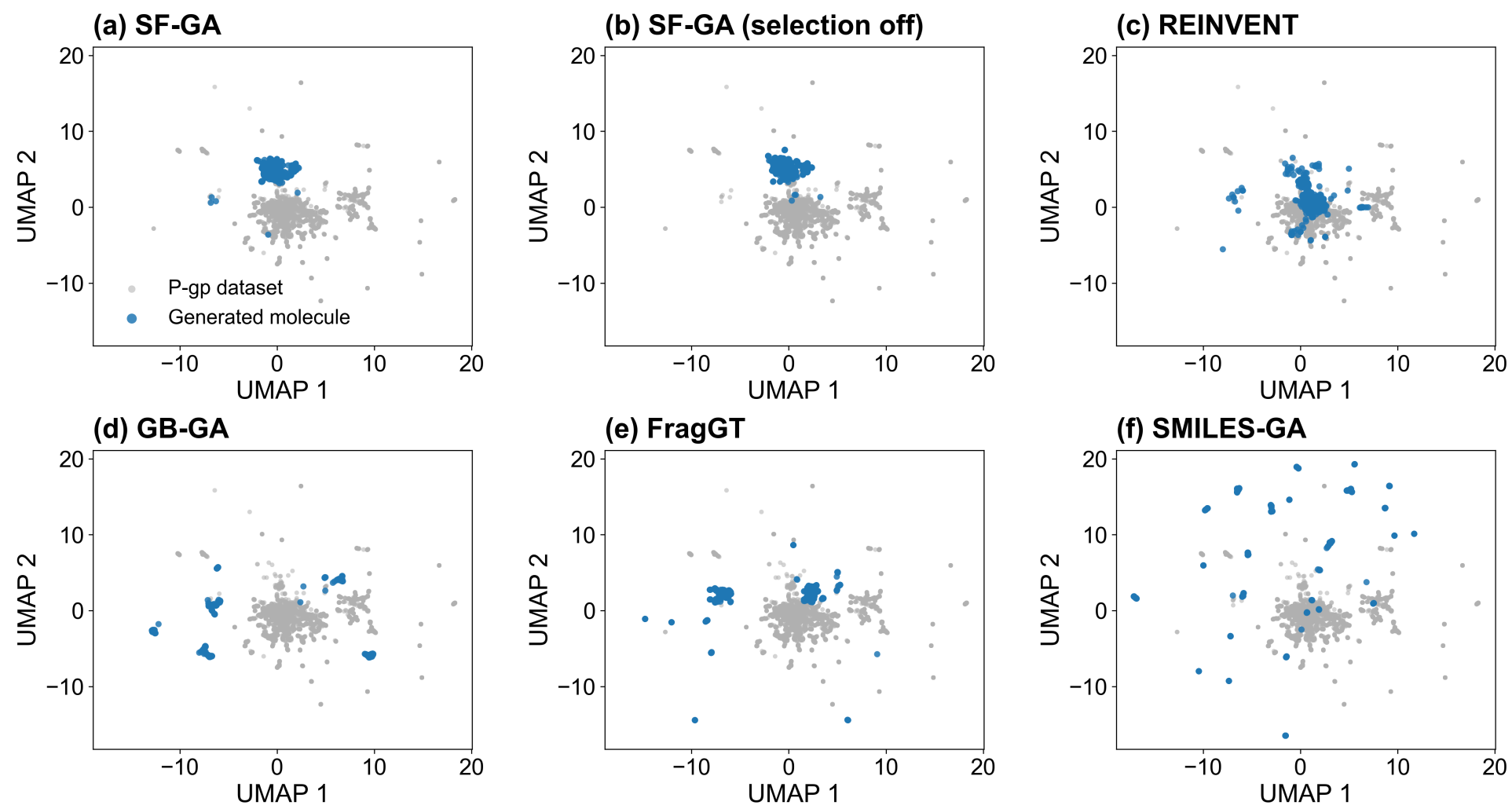

Figure S4: Chemical distributions of P-gp dataset (gray) and generated molecules (blue). Each panels are represented for generated molecules from models.

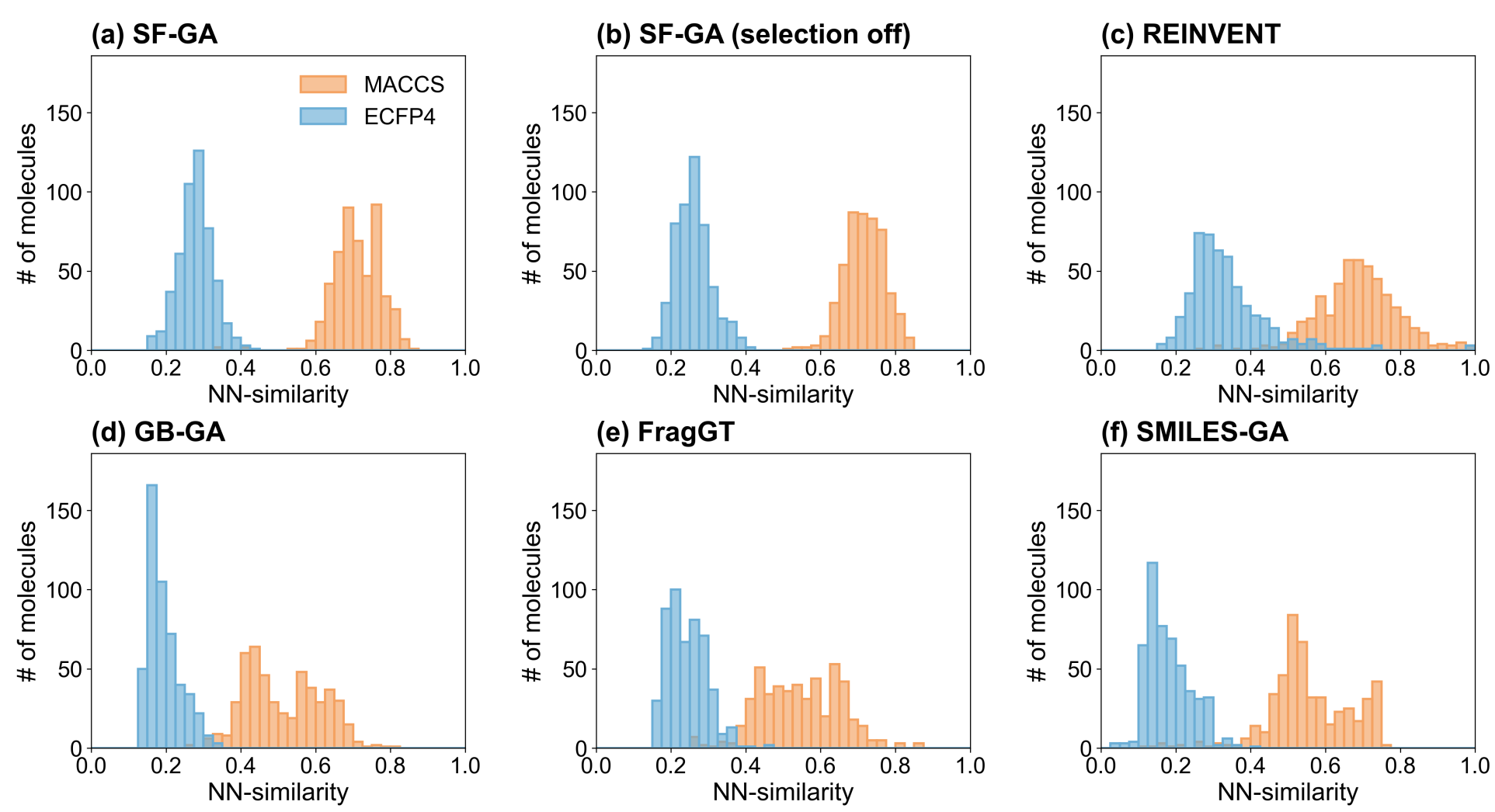

Figure S5: Tanimoto similarity distributions using MACCS (orange) and ECFP4 (blue) between P-gp dataset and generated molecules. Each panels are represented for generated molecules from models.

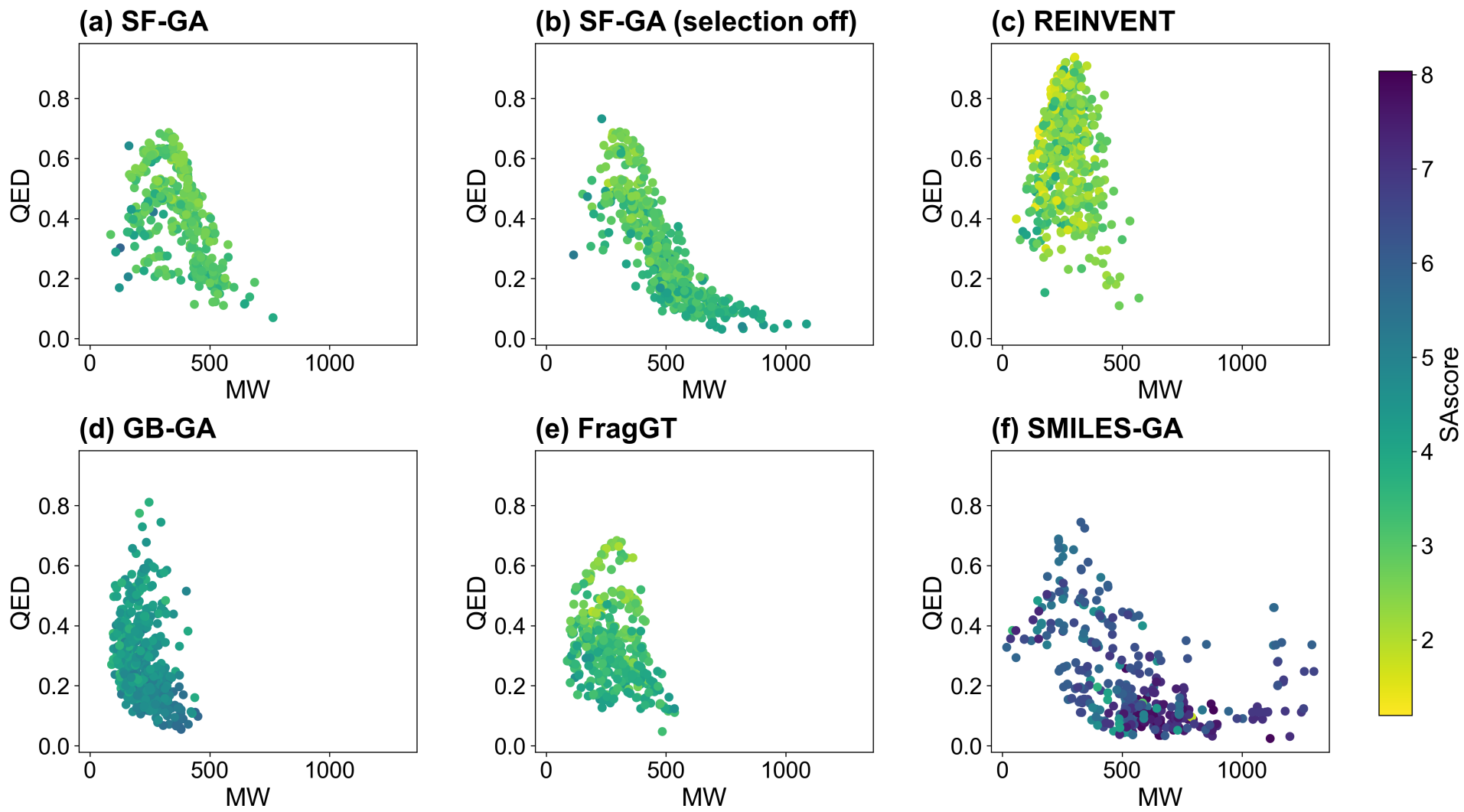

Figure S6: Distribution of molecular weight and QED for molecular sets generated by baselines: (a) SF-GA (this work), (b) SF-GA (selection-off), (c) REINVENT, (d) GB-GA, (e) FragGT, (f) SMILES-GA. Each point represents a molecule, and color indicates its synthetic accessibility (SA) score.

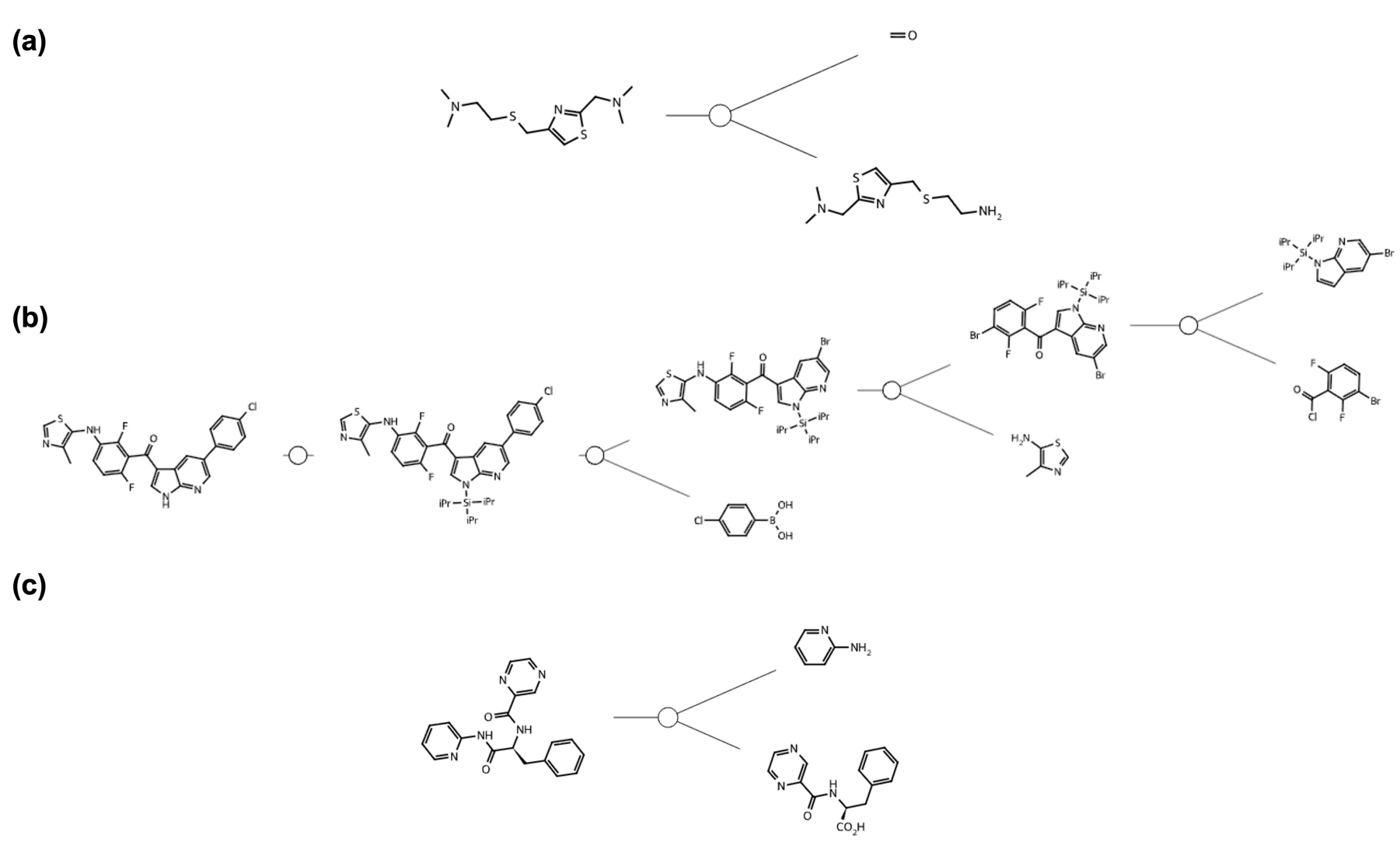

Figure S7: Retrosynthesis route for generated molecules. Each panels is mapped with each generated molecules in Figure 6.

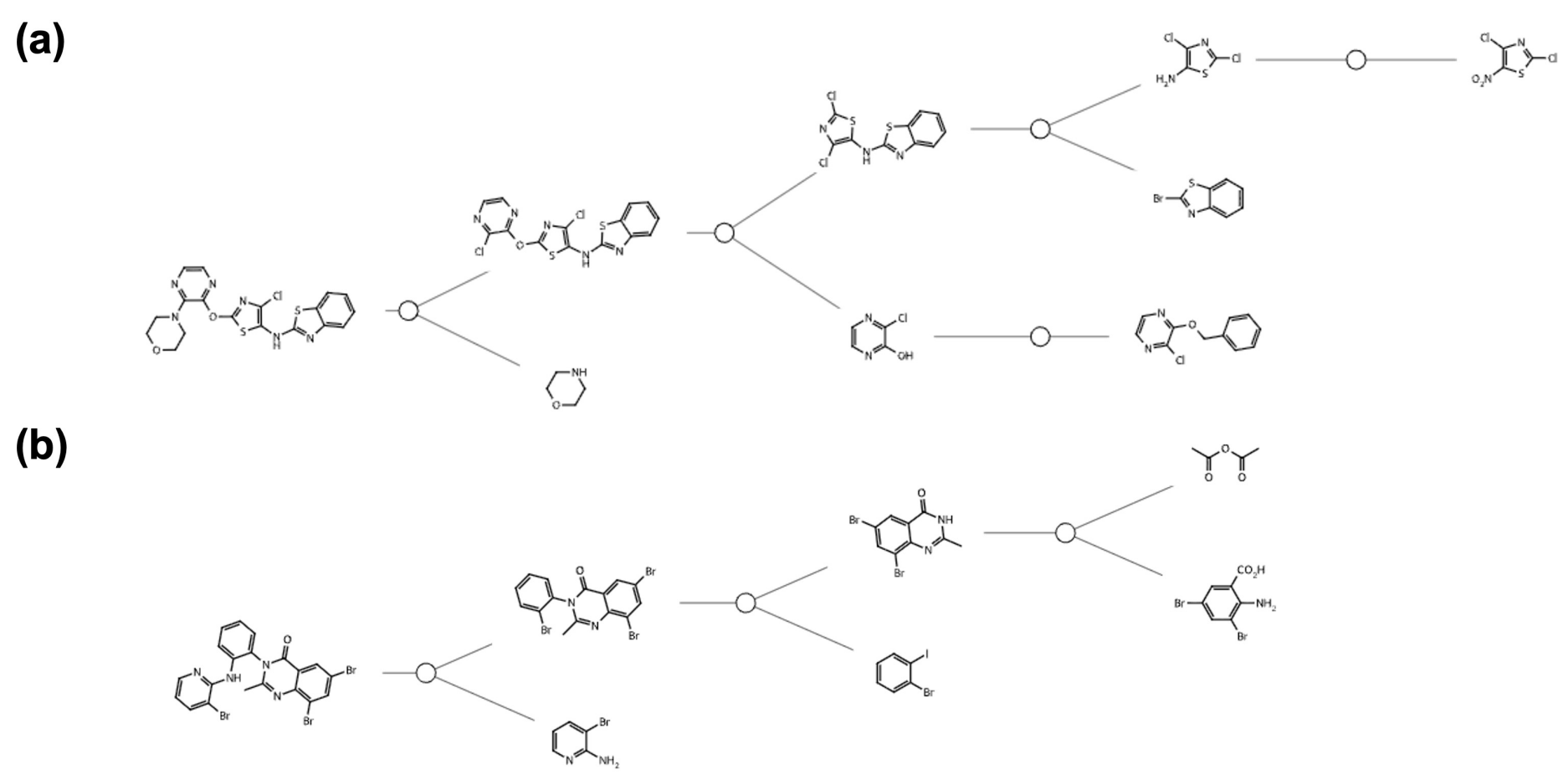

Figure S8: Retrosynthesis route for generated molecules. Each panels is mapped with each generated molecules in Figure 7.

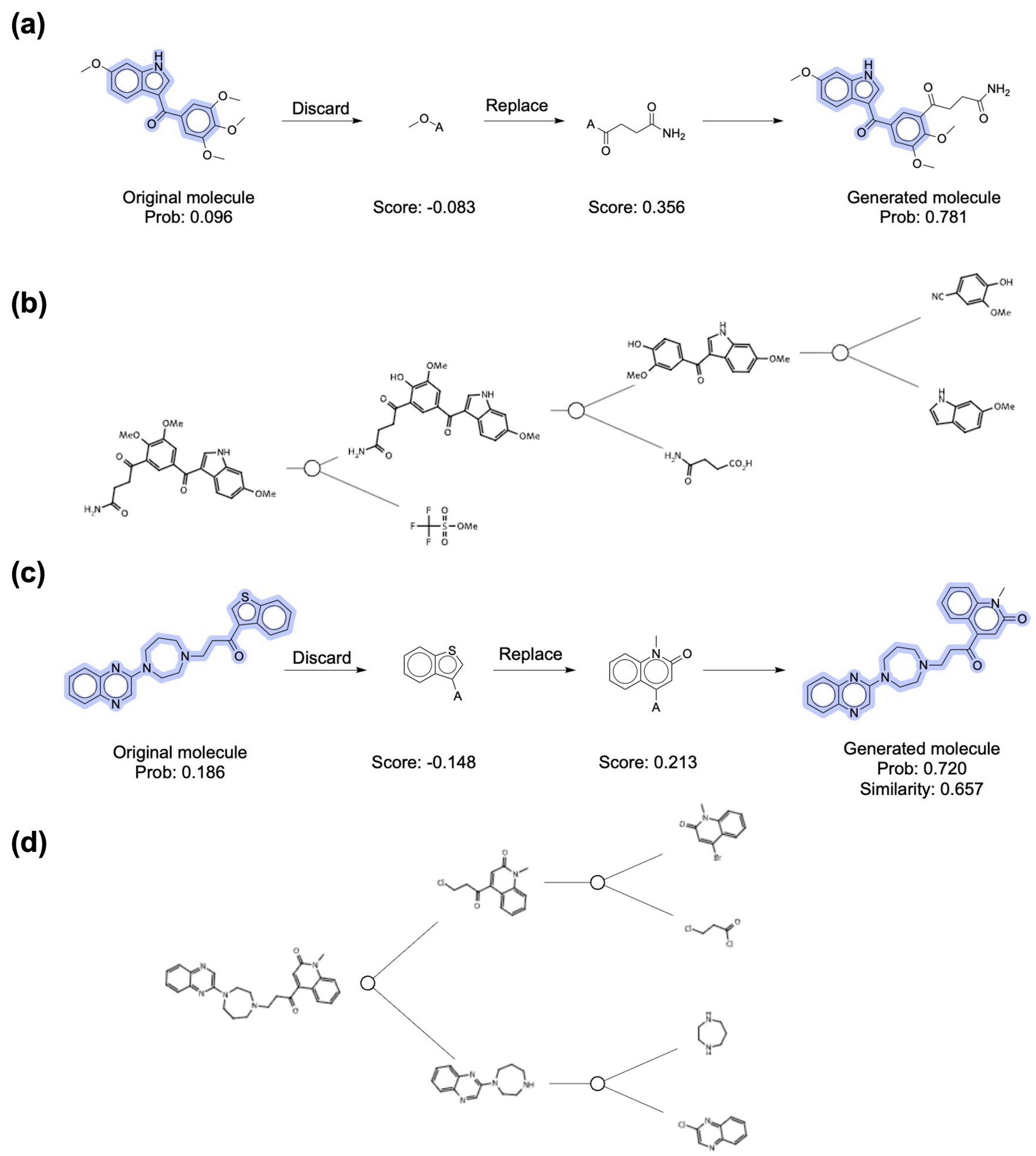

Figure S9: Examples of molecular editing for two P-gp non-substrate molecules. (a) is peripheral editing simulation and (b) is high-similarity editing simulation while (b) and (d) are represented for retrosynthetic route for generated molecules of (a) and (c) respectively.

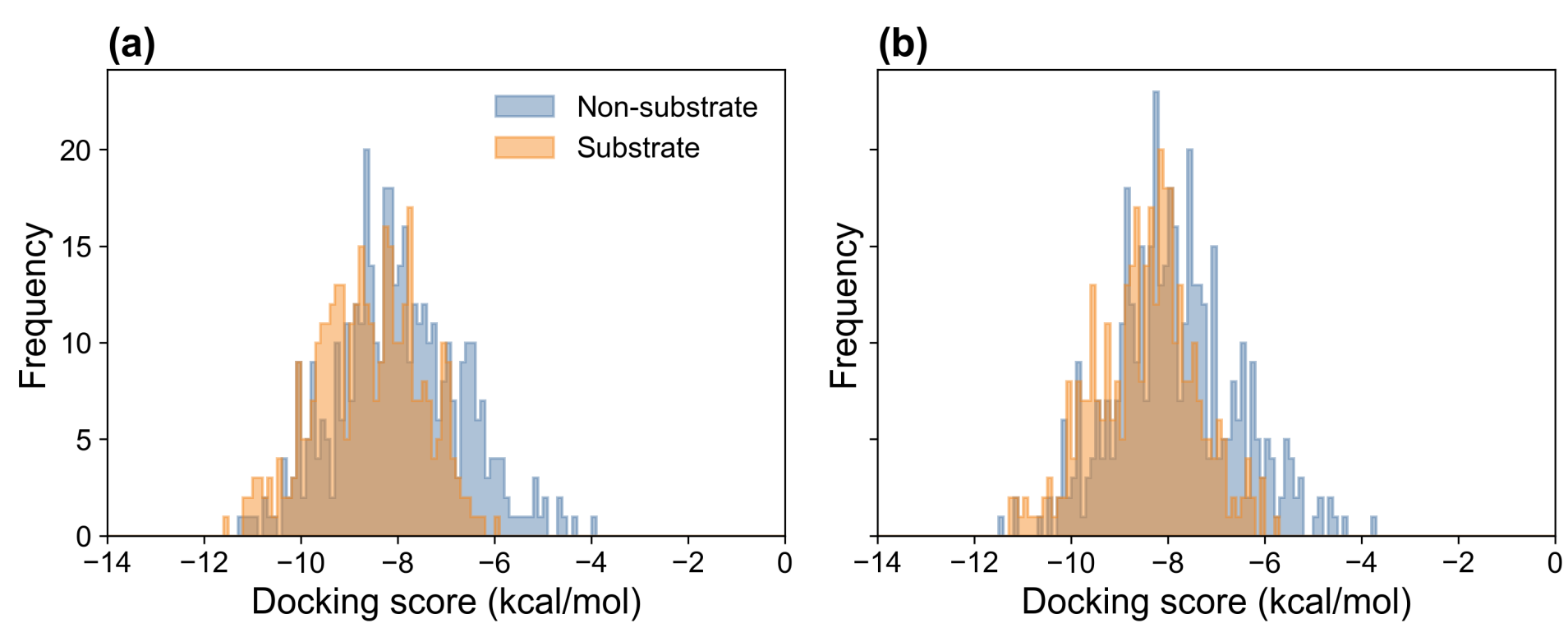

Figure S10: Docking score comparison between non-substrates and substrates in P-gp data.

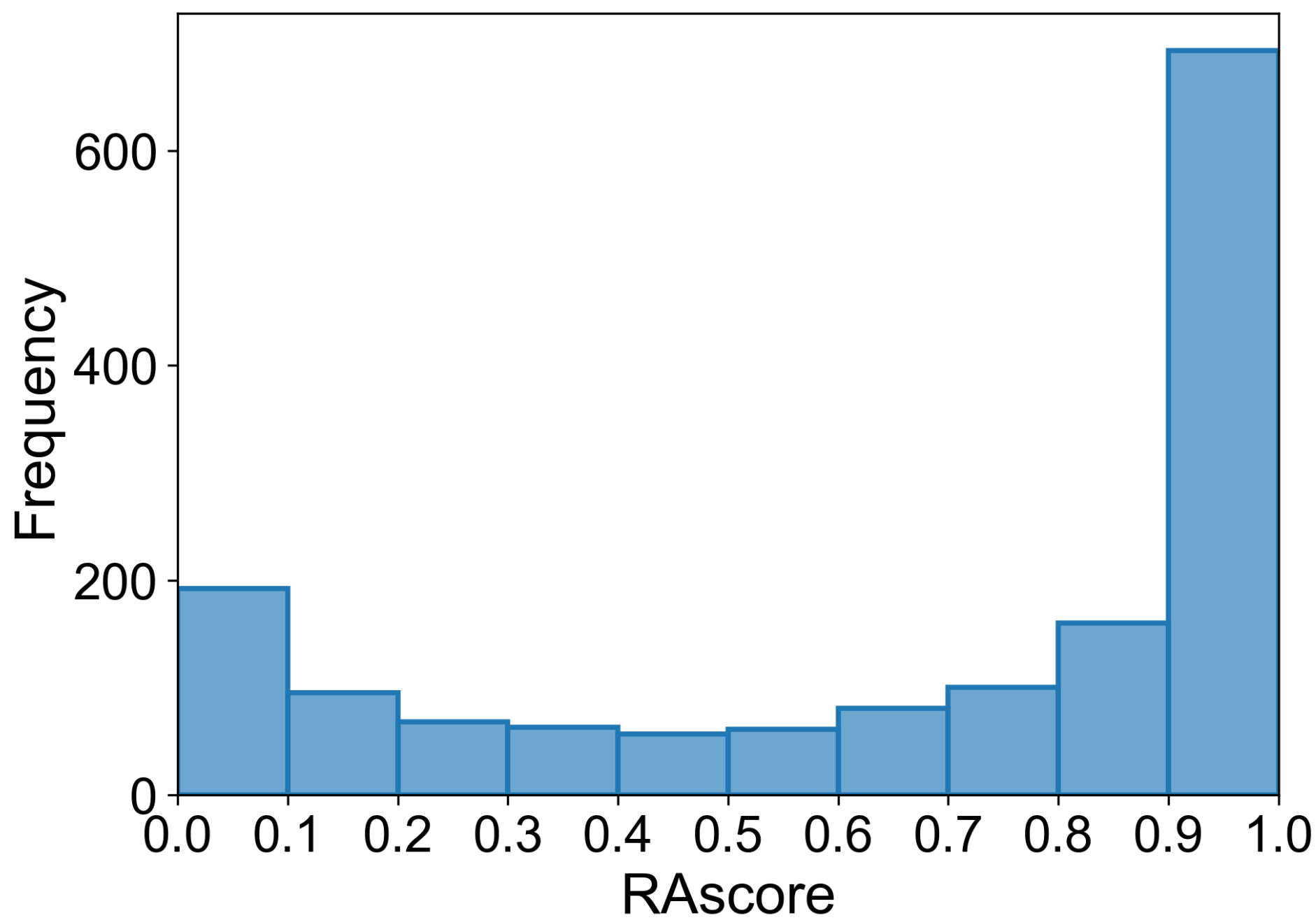

Figure S11: RA score distribution of 1,570 molecules generated by SF-GA after PAINS, NIH, and BRENK filtering.

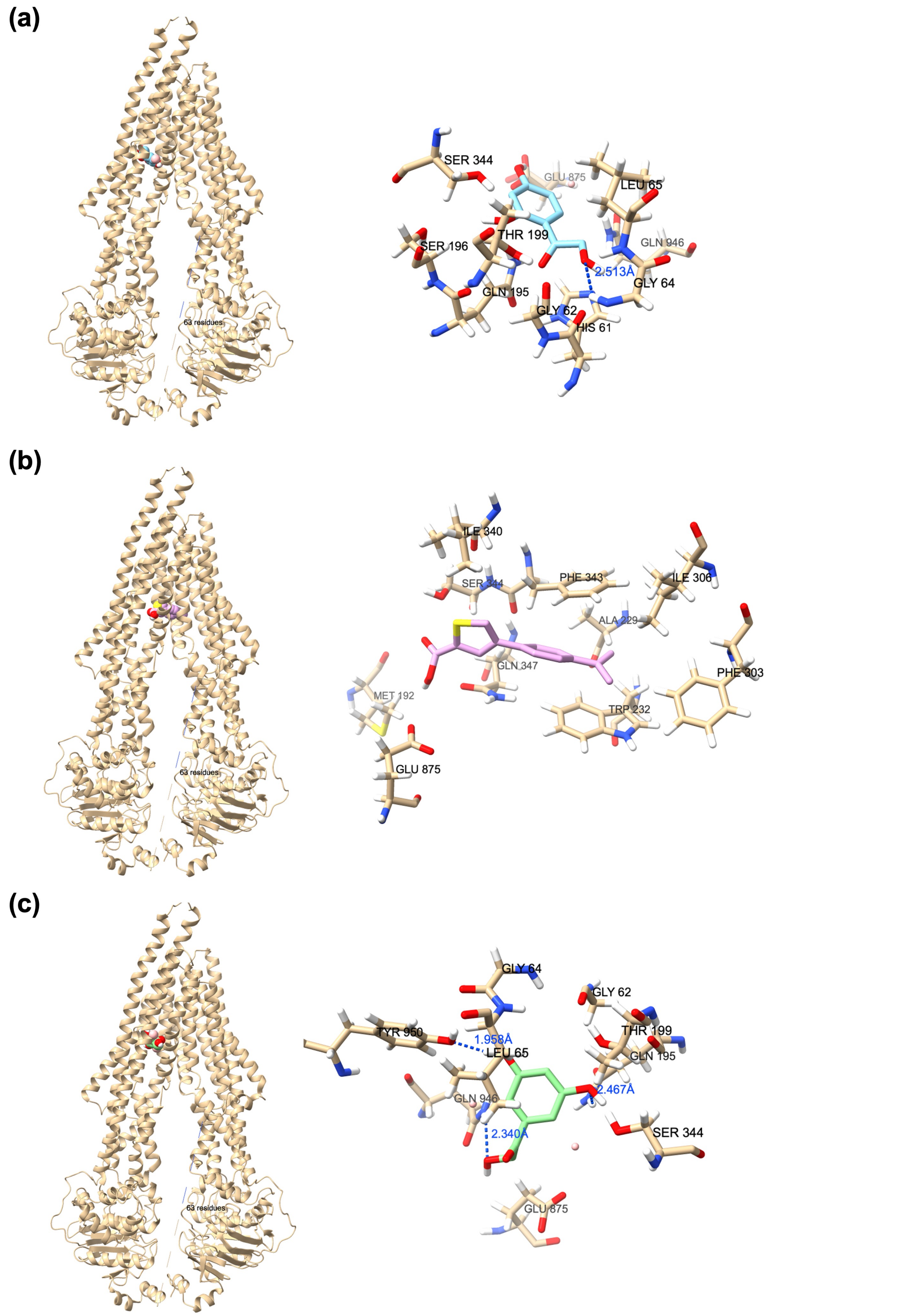

Figure S12: The first binding modes of ligands, including (a) ligand 1, (b) ligand 2, and (c) ligand 3, in P-gp (PDB ID: 6QEX). Detailed views of each ligands were right side image while residues within 3.5 Ã̃of the ligands and hydrogen-bonding interactions (color: blue).

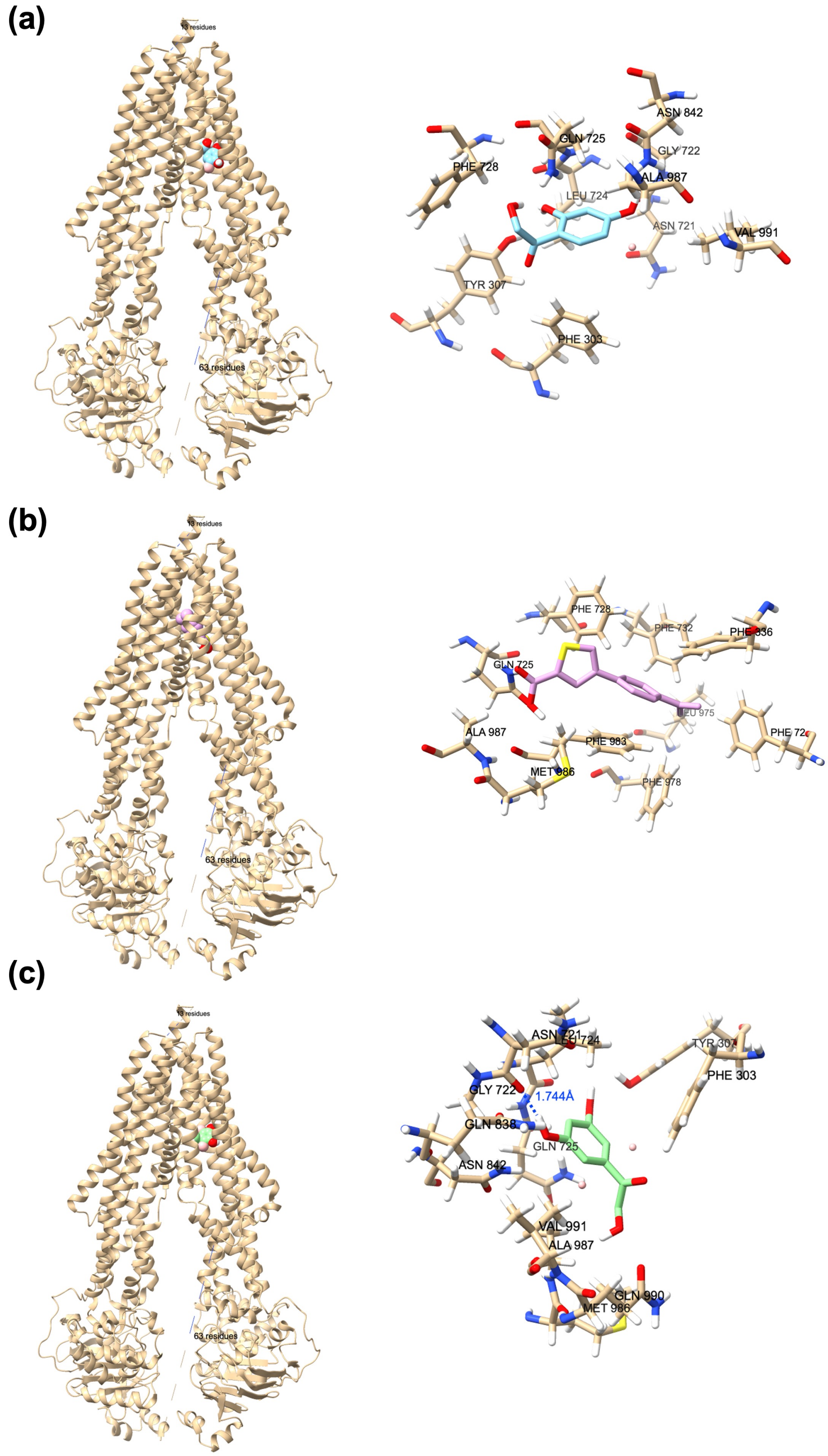

Figure S13: The first binding modes of ligands, including (a) ligand 1, (b) ligand 2, and (c) ligand 3, in P-gp (PDB ID: 7O9W). Detailed views of each ligands were right side image while residues within 3.5 Åof the ligands and hydrogen-bonding interactions (color: blue).
